# ER stress-induced aggresome trafficking of HtrA1 protects against proteotoxicity

**DOI:** 10.1093/jmcb/mjx024

**Published:** 2017-08-10

**Authors:** Maximilian J Gerhardt, Joseph A Marsh, Margaux Morrison, Andrius Kazlauskas, Arogya Khadka, Stephan Rosenkranz, Margaret M DeAngelis, Magali Saint-Geniez, Sarah Melissa P Jacobo

**Affiliations:** 1 Department of Ophthalmology, Harvard Medical School, The Schepens Eye Research Institute and Massachusetts Eye and Ear Infirmary, Boston, MA 02114, USA; 2 Department III of Internal Medicine, Cologne University Heart Center, Center for Molecular Medicine, University of Cologne, 50931 Cologne, Germany; 3 MRC Human Genetics Unit, Institute of Genetics and Molecular Medicine, University of Edinburgh, Edinburgh EH4 2XU, UK; 4 Department of Ophthalmology and Visual Sciences, University of Utah and John A. Moran Eye Center, Salt Lake City, UT 84132, USA

**Keywords:** ER stress, unfolded protein response, RPE, HtrA1, multi-domain protein evolution, proteostasis

## Abstract

High temperature requirement A1 (HtrA1) belongs to an ancient protein family that is linked to various human disorders. The precise role of exon 1-encoded N-terminal domains and how these influence the biological functions of human HtrA1 remain elusive. In this study, we traced the evolutionary origins of these N-terminal domains to a single gene fusion event in the most recent common ancestor of vertebrates. We hypothesized that human HtrA1 is implicated in unfolded protein response. In highly secretory cells of the retinal pigmented epithelia, endoplasmic reticulum (ER) stress upregulated HtrA1. HtrA1 co-localized with vimentin intermediate filaments in highly arborized fashion. Upon ER stress, HtrA1 tracked along intermediate filaments, which collapsed and bundled in an aggresome at the microtubule organizing center. Gene silencing of HtrA1 altered the schedule and amplitude of adaptive signaling and concomitantly resulted in apoptosis. Restoration of wild-type HtrA1, but not its protease inactive mutant, was necessary and sufficient to protect from apoptosis. A variant of HtrA1 that harbored exon 1 substitutions displayed reduced efficacy in rescuing cells from proteotoxicity. Our results illuminate the integration of HtrA1 in the toolkit of mammalian cells against protein misfolding and the implications of defects in HtrA1 in proteostasis.

## Introduction

The high temperature requirement A (HtrA) family consists of highly conserved, multi-domain proteins that are present in all kingdoms of cellular life (Clausen et al., 2002). Their importance is underscored by the identification of developmental (Hara et al., 2009; Tiaden et al., 2012a) or age-associated (Kooistra et al., 2009) human disorders linked to HtrAs, making this gene family a fertile ground for drug discovery. In recent years, human high temperature requirement A1 (HtrA1) has been the subject of drug discovery efforts in age-related macular degeneration (AMD) (Ciferri et al., 2015), owing to the prevalence of high-frequency *HTRA1* variants in affected subjects (Dewan et al., 2006; Yang et al., 2006). However, its precise role in AMD pathology remains controversial (Grassmann et al., 2017).

The signature protein architecture of HtrA family, which consists of a core serine protease domain appended at the C-terminus by a postsynaptic density 95/*Drosophila* disc large tumor suppressor/zonula occludens-1 (ZO-1) (PDZ) domain, performs dual peptide refolding and degradative chaperone (Malet et al., 2012) functions by oligomeric assembly (Krojer et al., 2008). Bacterial HtrAs detoxify misfolded proteins from various stressors (Lipinska et al., 1990; Johnson et al., 1991; Yamamoto et al., 1996). Pairwise comparisons of protein sequences from human vs. prokaryotic HtrAs show remarkably high (<33%) amino acid sequence identity and supports a case in favor of ancestral protein functional conservation throughout evolution. This conclusion, however, has been elusive for human HtrAs. Part of the difficulty is the emergence of N-terminal domains that resemble IGF-binding protein (IGFBP)-like and TGFβ-binding-like Mac25 and Kazal-type inhibitor (KI) proteins in human HtrA1, HtrA3, and HtrA4, differentiating them from other members of the family and raising the question how these new domains might alter protein function. The structure for full-length human HtrA1 at ~10 Å resolution places some ambiguity in the relationship between the N-terminal and core domains (Eigenbrot et al., 2012), and the available crystal structures of human HtrA1 truncate the N-domains (Truebestein et al., 2011; Eigenbrot et al., 2012).

Current views of the human HtrA set ascribe discrete functions to the N-domains, often independent from the core. HtrA1, HtrA3, and HtrA4 are believed to be secreted proteases. The N-terminal half are assigned growth factor binding functions (Oka et al., 2004; Tocharus et al., 2004; Hou et al., 2007; Kim et al., 2012; Jacobo et al., 2013). The core domains are implicated in remodeling of the extracellular matrix (Tiaden et al., 2012b). The catalytic serine protease domain, unlike that in prokaryotes, does not appear to rely on the PDZ domain for substrate capture (Truebestein et al., 2011; Eigenbrot et al., 2012). Presently, the challenge in the field is to demonstrate whether human HtrAs have dual protease-chaperone function in living cells, how the N-domains may influence this, and importantly, what consequences this may have to cell fate.

In an effort to fill the gaps in our understanding of this multi-domain protein family, we traced the domain accretion of Mac25 and KI as a single unit by an ancestral HtrA in the early vertebrate lineage. We combined this with biochemical analyses and focused on HtrA1 in the highly secretory cells of the retinal pigment epithelia (RPE). We report that HtrA1 was induced upon chronic proteotoxicity as part of the unfolded protein response (UPR). We found that HtrA1 co-aligned with vimentin intermediate filaments (IFs), and upon endoplasmic reticulum (ER) stress, trafficked to the aggresome at the microtubule organizing center (MTOC). HtrA1 knockdown in the face of proteotoxicity was deleterious to adaptive UPR, and concomitantly resulted in cell death. This was rescued by gene augmentation with HtrA1, but not by variants that lacked serine protease or harbored deleterious substitutions within the Mac25 domain. Collectively, our work unravels an intracellular pro-survival role for HtrA1 in proteome homeostasis and illuminates design requirements for strategies that target HtrA1.

## Results

### HtrA innovations in the chordate lineage

Multi-domain proteins constitute ~80% of the total collection of eukaryotic proteins (Buljan et al., 2010). Illuminating mechanisms of domain combination to produce signature architectures is rudimentary to our understanding of proteomes and evaluating functional consequences of disease-associated variations in the human genome.

The HtrA family of proteins are widespread in bacteria and eukaryotes, although largely absent from archaea (Koonin et al., 2008). Phylogenetic analysis suggested a mitochondrial origin for the eukaryotic HtrAs (Koonin and Aravind, 2002). A previous evolutionary study of Mac25-containing proteins in vertebrates placed the HtrA family in an unrelated clade from the IGFBPs, despite the fact that HtrA1, HtrA3, and HtrA4 share the same N-terminal Mac25 and KI domains (Rodgers et al., 2008). Aside from these existing reports, there is a gap in our understanding of when and how these N-domains were acquired by the HtrA family.

**Figure 1:**
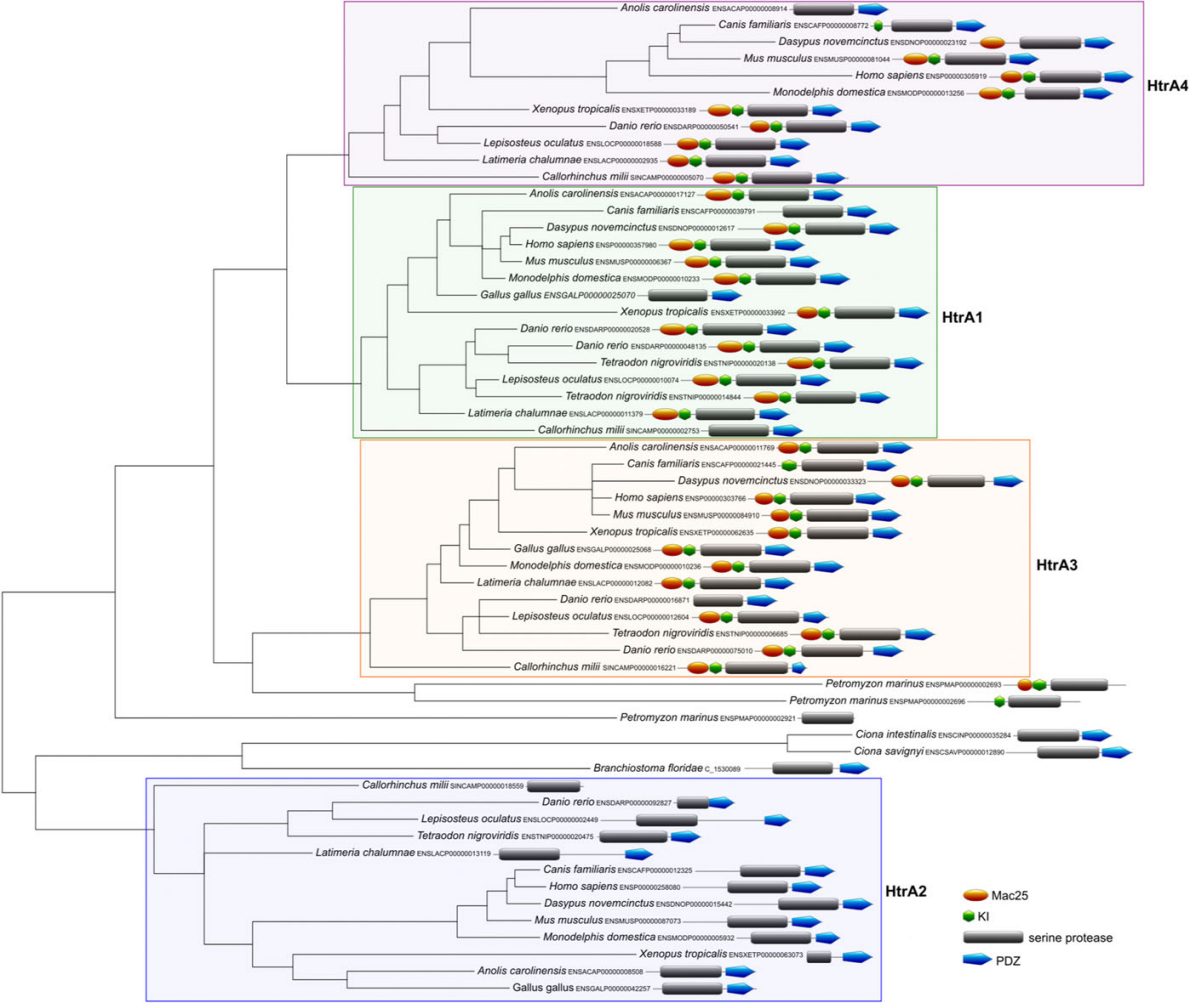
Molecular evolution of the chordate HtrA set.

An initial search for HtrA homologs showed that the core serine protease and PDZ domains are observed across essentially all eukaryotes. However, there were no HtrA proteins with N-terminal Mac25 or KI domains in any species outside the vertebrates. Therefore, in order to trace the evolution of the HtrA domain architecture, we performed a phylogenetic analysis of HtrA proteins from a diverse set of chordate genomes (Figure 1). We can see that the invertebrate chordates, *Ciona* (tunicate) and *Branchiostoma* (cephalochordate), each have just a single HtrA protein lacking the N-terminal domains. *Petromyzon* (lamprey) is the most distant species from mammals where HtrA proteins with N-terminal domains are observed. The proteins used for multiple sequence alignment are provided in Supplementary Dataset S1.

Most of the vertebrate HtrA sequences cluster into four clear families, reflecting the HtrA1−4 sequences present in humans. HtrA1, HtrA3, and HtrA4 all possess the N-terminal domains, with HtrA1 and HtrA4 being most closely related. Interestingly, the two *Petromyzon* sequences with N-terminal domains are more closely related to each other, but do not cluster with other vertebrate HtrA proteins. Thus, we can infer the likely evolutionary origin of these families. First, there was a gene duplication of an ancestral HtrA gene. One of those copies is the ancestor of the HtrA2 family, while the other underwent a gene fusion event to acquire the N-terminal Mac25 and KI domains. This domain acquisition had occurred by the time of the most recent common ancestor of the vertebrates. Following this, there were two more gene duplication events, first with HtrA3 diverging, and then divergence between HtrA1 and HtrA4. Both of these duplications must have occurred by the time of our most recent common ancestor with the cartilaginous fish, since members of all four families are present in *Callorhinchus* (elephant shark).

In the invertebrate chordate species *Ciona intestinalis*, where HtrA has not acquired the N-domains, there are two other proteins (ENSCINP00000012789 and ENSCINP00000016757) that contain N-terminal Mac25 and KI domains, followed by a C-terminal immunoglobulin domain, suggesting that they are homologs of the human Kazald1, IGFBP7 (Mac25) and IGFBPL1 proteins (Rodgers et al., 2008). This also suggests that the Mac25 and KI domains were acquired together via a single evolutionary gene fusion event from one of the ancestral IGFBP-like proteins.

Figure 1 also shows a few peculiarities in terms of domains absent from some sequences. For example, all three *Petromyzon* sequences and the *Callorhinchus* HtrA2 lack C-terminal PDZ domains. Furthermore, one of the *Petromyzon* sequences lacks an N-terminal Mac25 domain, but does have a KI domain, while a few of the HtrA1, HtrA3, and HtrA4 sequences from other species lack one or both N-terminal domains. It is difficult to say whether these represent genuine evolutionary changes in domain architecture, or whether they are due to genome annotation errors. The *Petromyzon* genome in particular is incredibly difficult to analyze, due to its high GC content, large amount of repetitive elements, and the lack of genome sequences for any closely related species (Smith et al., 2013). Notably, the exon encoding the N-terminal domains has exceptionally high GC content. Thus, genome annotation errors involving these domains would not be surprising.

Evolution of proteins by domain acquisition can substantially alter protein function by proving new activities (Rodgers-Melnick et al., 2013) and modulating the structure of pre-existing domains (Marsh and Teichmann, 2014, 2015). We next aimed to test whether gains in N-domains influence chaperone-protease functions in human HtrAs. The case of HtrA1 is interesting, as all AMD-associated HtrA1 variants identified thus far reside within the promoter region upstream of the accrued exon that encodes the N-domains, and within this exon itself.

### ARPE-19 cells upregulate HtrA1 upon chronic protein misfolding

**Figure 2:**
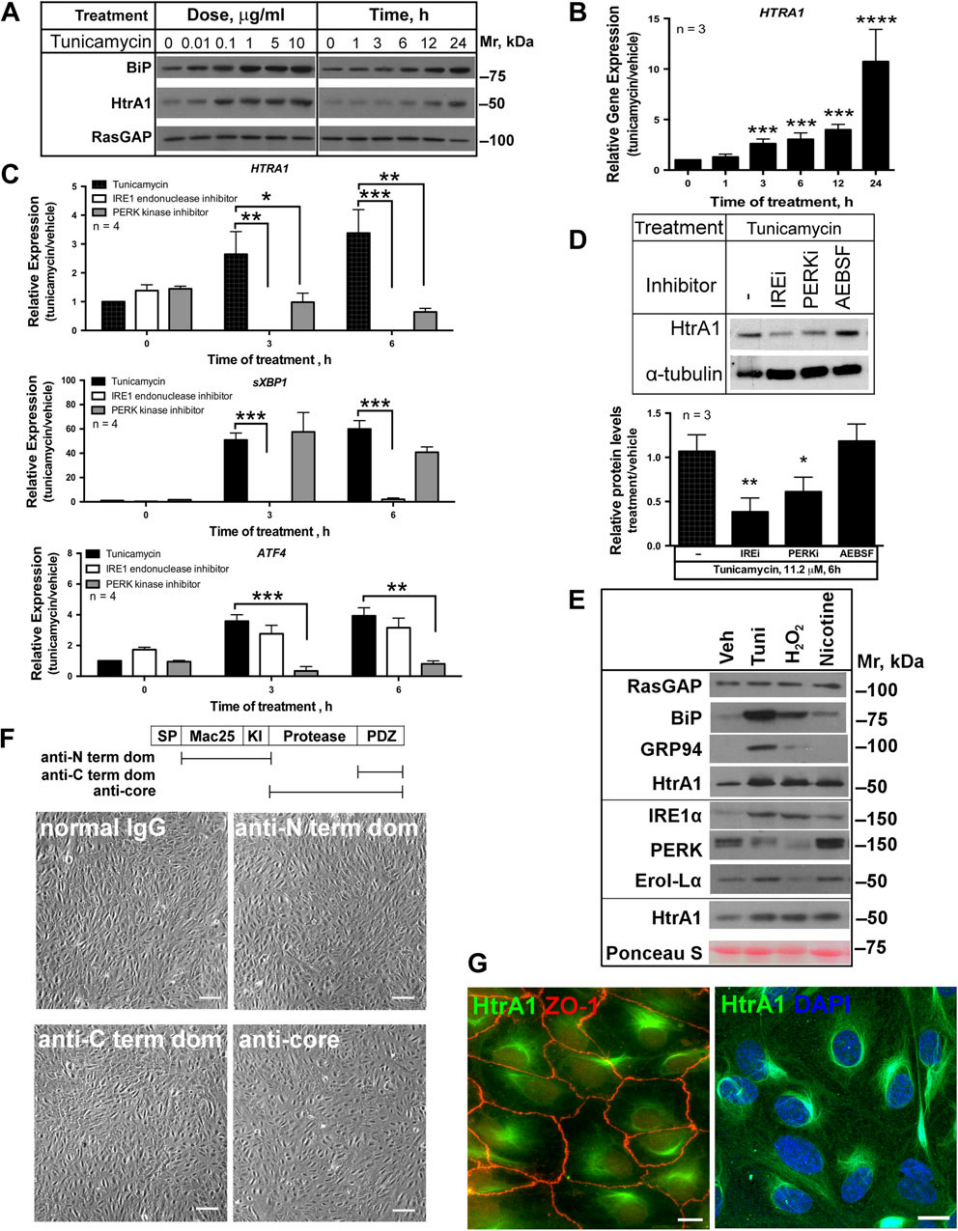
RPE cells upregulate HtrA1 during ER UPR. (**A**) Dose- and time-dependent upregulation of HtrA1 protein after tunicamycin-induced proteotoxicity. Induction of ER stress was validated by probing for BiP (positive control). Ras GTPase-activating protein (RasGAP) served as loading control. Immunoblots are representative of *n* > 3 experiments. (**B**) HtrA1 transcript was upregulated upon ER stress. Relative gene expression was calculated with 0 h as reference. ***P < 0.001, *n* = 3. (**C**) Blockers of IRE1α ribonuclease (4μ8C, 100 nM) or PERK (GSK2606414, 10 nM) suppressed HtrA1 mRNA upregulation upon ER stress induced with tunicamycin (11.2 μM). sXBP-1 (IRE1α target) or ATF4 (PERK target) was used as positive control. *P < 0.05, *n* = 4. (**D**) Blockers of UPR prevented HtrA1 protein upregulation upon ER stress. Immunoblot validation of HtrA1 gene expression analyses in **C**. *P < 0.05, *n* = 3. (**E**) Cells upregulated HtrA1 after overnight treatment with oxidizing stressor H_2_O_2_ (0.5 mM) or nicotine (2 μM). Immunoblots are representative of *n* > 3 experiments. (**F**) Sequestration of exported HtrA1 with neutralizing antibodies did not compromise ARPE-19 cell viability. ARPE-19 cells were stimulated with tunicamycin (5.62 μM). After 6 h, anti-HtrA1 antibodies against the N-domain, the core domain, or the C-terminal domain were added to the conditioned media. The final concentrations were 208−533× in molar excess to the amount of HtrA1 typically exported by ARPE-19 within 24 h ER stress. After additional 18 h of incubation (in total 24 h treatment), brightfield images were collected to monitor overt signs of toxicity. Images are representative of three random fields per coverslip (*n* = 3). Scale bar, 100 μm. (**G**) Intracellular localization of HtrA1 in ARPE-19 cells. Cells were immunostained with monoclonal anti-HtrA1 antibody, and counterstained with ZO-1 or DAPI. scale bar, 10 μm.

We set up conditions in cells so that their requirement for chaperones is a matter of life or death. Using cultured RPE (ARPE-19) as a model, we mimicked conditions wherein immature, misfolded proteins accumulate by blocking the ER-to-Golgi translocation of nascent proteins with the N-glycosylation inhibitor tunicamycin (Kuo and Lampen, 1974), and thereby trigger ER stress (Feige and Scheffler, 1987). Immunoblot analyses of total ARPE-19 lysates verified the dose- and time-dependent activation of ER stress with a marker, the UPR master regulator immunoglobulin heavy chain binding protein (BiP)/GRP78 (glucose-responsive protein, 78 kDa) (Munro and Pelham, 1986). We found that HtrA1 was upregulated in response to chemical induction of ER stress (Figure 2A). Increased HtrA1 translation was also accompanied by enhanced HtrA1 transcription (Figure 2B).

BiP is a binding partner and tonic inhibitor of UPR effectors that reside at the ER membrane: protein kinase RNA-like ER kinase (PERK), activating transcription factor 6 (ATF6), and inositol-requiring enzyme (IRE). We validated whether these arms of UPR were active in ARPE-19 (Supplementary Figure S1). qRT-PCR analyses showed that the downstream transcription factor targets of PERK, activating transcription factor 4 (*ATF4*) and DNA damage inducible transcript 3 (*DDIT3*), were significantly enhanced after 1 h of tunicamycin exposure. *ATF4* returned to baseline by 3 h, and *DDIT3* expression was sustained up to 24 h. Tribbles homolog 3 (*TRIB3*), which encodes a transcription factor that antagonizes CCAT/enhancer-binding protein homologous protein (CHOP; gene product of *DDIT3*), was upregulated at 3 h and sustained through 24 h chemical ER stress induction. ATF6 and IRE targets were upregulated as of 3 h.

We next asked whether the ER stress-induced HtrA1 upregulation depended on the gene regulation network governed by BiP-dependent mediators. We found that after 6 h of chemically induced ER stress, HtrA1 transcription was abolished by 4μ8C, a small molecule blocker of IRE1α’s ribonuclease subunit (Cross et al., 2012). A Ser/Thr kinase inhibitor of PERK, GSK2606414 (Axten et al., 2012), only partially blocked HtrA1 transcription (Figure 2C). Neither of these inhibitors significantly affected the transcription of HtrA1, spliced Xbox-binding protein 1 (sXBP-1), or ATF4 in the absence of chemically induced ER stress. Immunoblot analyses of ARPE-19 lysates validated that suppression of HtrA1 transcription by inhibiting IRE1α or PERK correlated with diminished HtrA1 protein. These observations support the conclusion that HtrA1 is upregulated during UPR in a manner that depended on IRE1α and PERK (Figure 2D).

Previously, cultured RPE chronically exposed to cigarette smoke extract had an overrepresentation of UPR genes and persistently active IRE1α, PERK, and ATF6. Some of these features were found in RPE from early AMD human subjects (Cano et al., 2014). Given these antecedents and the enrichment of HtrA1 risk variants in a subset of AMD subjects (Supplementary Table S1), we were thus curious whether *in vitro* surrogates (Suner et al., 2004; Supanji et al., 2013) for these stressors induce HtrA1 expression in RPE. We found that overnight treatment (18 h) of ARPE-19 with H_2_O_2_ or nicotine upregulated HtrA1, albeit only weakly increased BiP and GRP94β (glucose-regulated protein, 94 kDa) (Figure 2E, top box). Nevertheless, the enhanced expression of HtrA1 upon prolonged exposure to H_2_O_2_ or nicotine resulted in upregulation of IRE1α and suppression of PERK (Figure 2E, middle box).

Adaptive UPR results in tightly regulated ER traffic (Wei et al., 2011). HtrA1 harbors a putative signal peptide but lacks glycosylation (Yin et al., 2013), and we were curious whether its export from secretory epithelia like RPE was affected during stress response. We collected 24-h conditioned media and the corresponding total cell lysates from RPE and probed for exported HtrA1 by immunoblot (Figure 2E, bottom box). Upon stress, the exit from cells was not suppressed, and HtrA1 export was enhanced relative to vehicle. Notably, the steady-state levels of HtrA1 retained in cells were at least 5–8-fold higher than that exported. This ratio of intracellular vs. exported HtrA1 was not robustly different from that previously observed in patient-derived lymphocytes or after heterologous expression in HEK293T (Jacobo et al., 2013).

We next inquired about the relative contribution of the exported vs. intracellular pools of HtrA1 to ER stress response in RPE. We estimated the amount of exported HtrA1 by immunoblot after resolving RPE conditioned media side by side with known quantities of HtrA1 standards, and administered a single bolus of excess anti-HtrA1 neutralizing antibodies as previously characterized (Jacobo et al., 2013). In the face of ER stress, sequestration of exported HtrA1 with anti-HtrA1 antibodies that target either the N-domains, the core domains, or just the PDZ domain did not appear to trigger ER-associated death (Figure 2F). We reasoned that the amount of secreted HtrA1, although higher than baseline, nonetheless constituted a small fraction of that packaged in secretory granules. We also accounted for the likelihood that intracellular HtrA1 may be stranded in endosomes and unable to escape to secretory granules in the face of ER stress. We probed for intracellular HtrA1 by immunostaining RPE with a monoclonal antibody raised against human HtrA1 core domains. Instead of detecting HtrA1 in secretory granules that are typically docked at punctae beneath the cell membrane, we found that HtrA1 distributed in arborized fashion, extending out to the cytoplasm from the perinuclear region (Figure 2G).

### HtrA1 associates with vimentin IFs and traffics to aggresome upon ER stress

**Figure 3:**
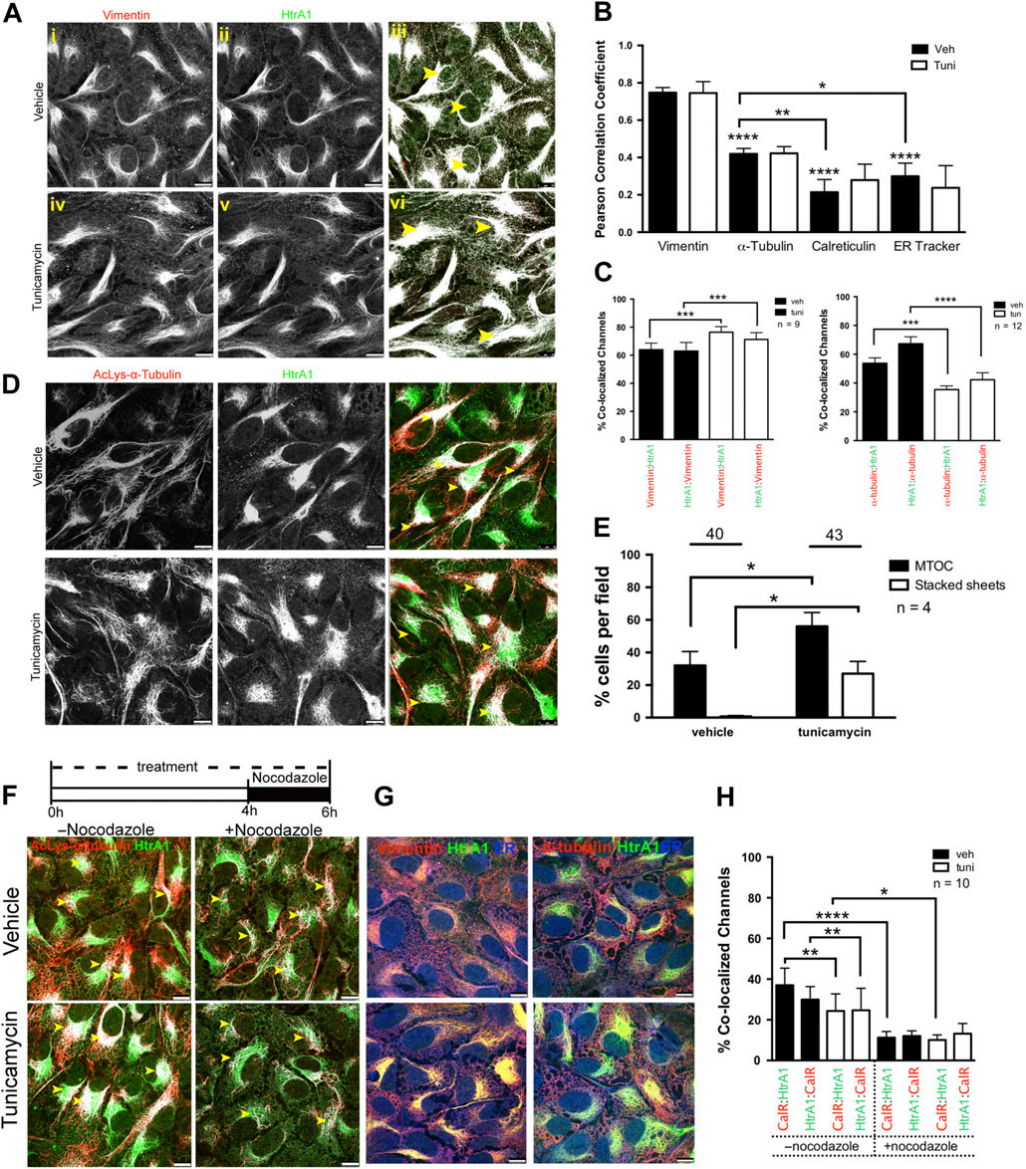
HtrA1 associates with vimentin IFs and traffics to the MTOC in the face of chronic proteotoxicity. (**A**) HtrA1 (green channel, panels ii, v) co-localized with vimentin IFs (red channel, panels i, iv) in vehicle-treated and ER-stressed ARPE-19 cells. In co-localized pixel maps (panels iii, vi), white areas show HtrA1 and vimentin pixel co-distribution (highlighted by yellow arrowheads). Scale bar, 10 μm. (**B**) Pearson correlation coefficients showed greatest co-occurrence of HtrA1 with vimentin IFs. *P < 0.05. (**C**) The percentage of co-localized channels was plotted after validating Mander’s correlation coefficient with Costes thresholding. *P < 0.05. (**D**) Immunostaining of Ac-lys40-α-tubulin (panels i, iv) and HtrA1 (panels ii, v) showed the bundling and enrichment at the MTOC upon ER stress. Scale bar, 10 μm. (**E**) HtrA1 was enriched at the MTOC after 6 h ER stress. We surveyed cells/field wherein HtrA1+ structures were selectively enriched at the MTOC (*P = 0.0246, paired *t*-test) or collapsed bundles (*P = 0.0257, paired *t*-test) and plotted the percentage of cells under resting or stressed conditions. Numbers above the bars indicate total number of surveyed fields (10−15 ROIs/field), encompassing *n* = 4 experiments. (**F** and **G**) De-stabilization of MTs and vimentin IFs attenuated HtrA1 trafficking to MTOC (**F**). Co-localized pixel maps showed diminished overlap of HtrA1 with Ac-MTs and IFs after nocodazole treatment (**G**). Yellow arrowheads point to channel co-localization examples. Images are representative of fields surveyed as described in **A**−**D**. Scale bar, 10 μm. (**H**) De-stabilization of MTs uncoupled HtrA1 and ER. The percentage of channel overlap of HtrA1 (green) with calreticulin (red) in vehicle-treated or stressed cells with nocodazole. *P < 0.05.

HtrA1 was previously detected in intracellular compartments, where it accomplished some proposed tasks, including association with microtubules (MT) (Chien et al., 2009) and proteolytic deactivation of tuberous sclerosis complex 2 (TSC2) (Campioni et al., 2010). In light of this and our observations on UPR signaling cascades that possibly integrate HtrA1, we hypothesized that HtrA1 associates with components of the cytoskeleton that are involved in ER structure and function (Lee et al., 1989) and remodeling in times of stress (Harding et al., 2003). We validated whether HtrA1 associates with MTs by co-staining HtrA1 with α-tubulin (Supplementary Figure S2A). In RPE cells, this co-localization was only partial and enriched around the nucleus. This correlated with partial overlap of HtrA1 with Calreticulin and ER Tracker at the perinuclear region (Supplementary Figure S2B). We did not observe significant HtrA1 staining when we replaced the anti-HtrA1 antibody with negative control (Supplementary Figure S3). Interestingly, we found that in RPE, HtrA1 fully co-aligned with vimentin IFs (Figure 3A and B, Supplementary Figure S3A and B).

Whereas HtrA1 appeared arborized in resting cells (Figure 2G), 6 h ER stress induction resulted in enrichment of HtrA1 in collapsed structures that selectively anchored at pericentriolar foci. In some cases, HtrA1 was exclusively bundled at this location (Supplementary Figure S3C). Vimentin IFs were remodeled in similar fashion (Figure 3A). Fractional overlap of HtrA1 with vimentin IFs shows that ER stress slightly enhanced their co-localization, likely due to upregulation of HtrA1. In contrast, ER stress reduced overlap of HtrA1 and α-tubulin, which possibly reflects the reduced surface area upon collapse of HtrA1+ structures and their enrichment at the pericentriolar foci (Figure 3C, Supplementary Figure S2C). These foci are consistent with the MTOC, and are enriched in nocodazole-resistant α-tubulin post-translationally modified with acetylation at Lys40 (Figure 3D). We quantified the number of cells/field wherein HtrA1 immunostaining were enriched at the MTOC (Figure 3E, black bars) and appeared as radially arborized or collapsed bundles (Figure 3E, white bars). We found significant enrichment of HtrA1 in the collapsed bundles at the MTOC in response to 6 h tunicamycin treatment.

These observations are reminiscent of aggresomes (Johnston et al., 1998), which assemble as part of a transient, reversible response to unfolded proteins that are typically marked for clearance by ubiquitination and proteasomal degradation (Ardley et al., 2003). Aggregates that cannot be degraded by ubiquitination inhibit the proteasome, and then transported to the MTOC and caged by vimentin IFs for clearance by autophagic lysosomes. When we de-stabilized MTs and IFs with nocodazole, we observed diminished intensity of HtrA1 in tunicamycin-treated cells. We also observed reduction, though not full inhibition, of HtrA1 bundling and targeting to pericentriolar foci, where it remained co-distributed in arborized fashion with unusually stable Ac-α-tubulin (Figure 3F, G and Supplementary Figure S3D). Finally, de-stabilization of MTs correlated with reduced overlap of HtrA1 with Calreticulin (Figure 3H).

These findings demonstrate the requirement for intact cytoskeletal IFs and MTs in the upregulation of HtrA1. Given that Ac-α-tubulin marks MT regions that serve as sites for rudimentary ER functions (Friedman et al., 2010), and that HtrA1 is induced by UPR effectors upon stress and thereafter traffics to aggresomes, we were next interested in the contribution of HtrA1 to the ER UPR.

### System-wide perturbations in ER UPR upon HtrA1 knockdown

**Figure 4:**
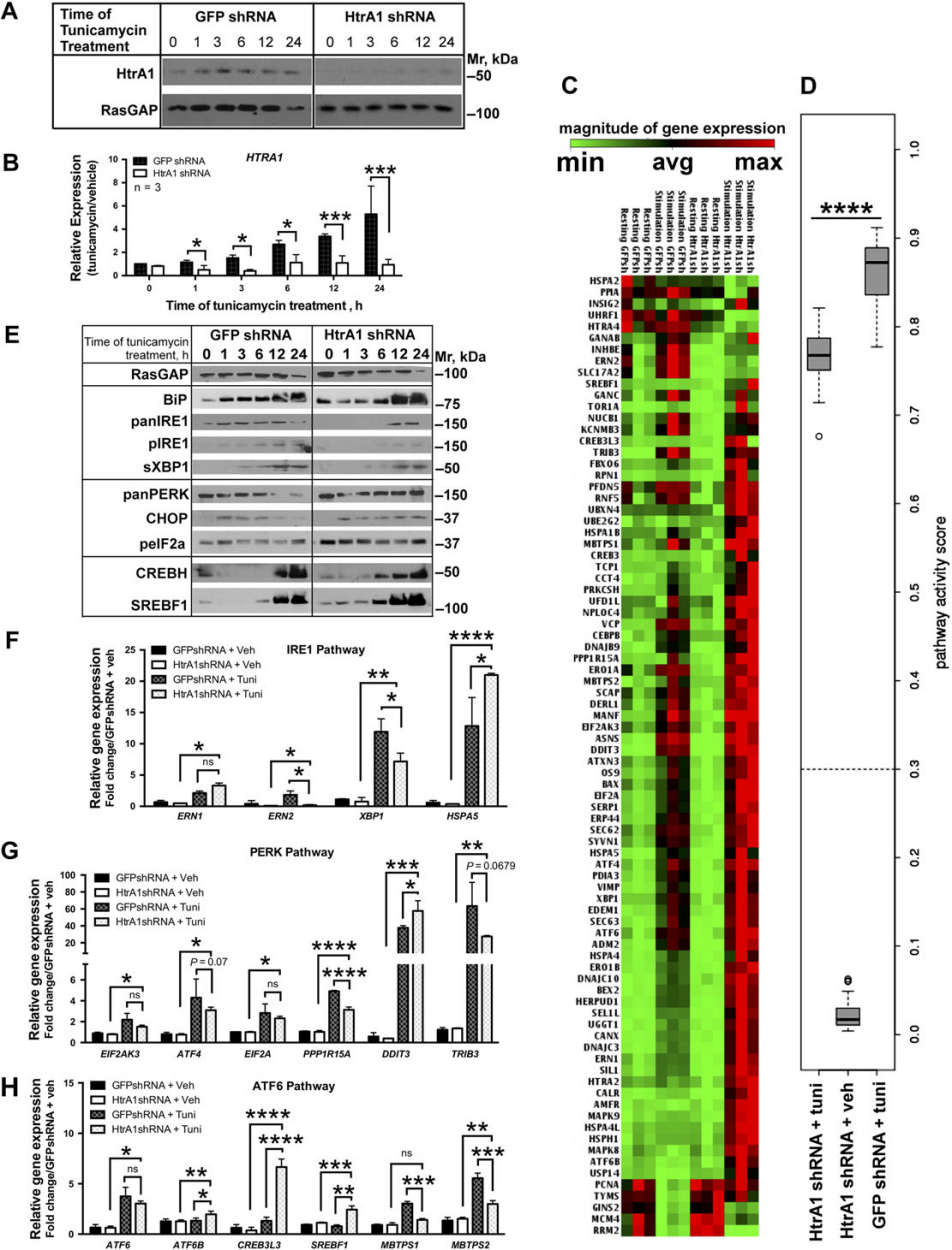
System-wide perturbation of ER UPR after HtrA1 ablation favors pro-apoptotic over adaptive signaling. (**A** and **B**) Immunoblot and real-time qRT-PCR validation of lentivirus-mediated delivery of human HtrA1 shRNA in RPE. (**C** and **D**) HtrA1 deficit in the face of chronic proteotoxicity resulted in system-wide changes in UPR transcripts. Average relative gene expression of analytes after 12 h tunicamycin (5.62 μM) treatment was shown as heat map (**C**). (**D**) Control vs. HtrA1-deficient cells displayed significantly different UPR pathway activation scores. ***P < 0.001, *n* = 3. (**E**) HtrA1 deficit in the face of proteotoxicity disrupted signal relay of IRE1, PERK, and ATF6. Total cell lysates were collected as described in **A** and **B** (*n* = 3 independent experiments). (**F**−**H**) Gene expression analysis of IRE1 (**F**), PERK (**G**), and ATF6 (**H**) pathway effectors. Total RNA was collected for gene expression analysis as in **C**. Relative mRNA levels were normalized to values from vehicle-treated GFP shRNA controls. *P < 0.05, *n* = 3.

We examined the contribution of intracellular HtrA1 to ER stress by stably expressing HtrA1 shRNA in ARPE-19 using lentivirus delivery. We accounted for the possibility that viral transduction itself can stimulate ER stress. In parallel to HtrA1 knockdown cells, we transduced parental ARPE-19 cells with lentivirus-encoded shRNA against the jellyfish green fluorescent protein (GFP) as non-HtrA1 silencing control. We validated the efficacy of knockdown and found that at a constant dose of tunicamycin, stable expression of the HtrA1 shRNA, but not the GFP shRNA, prevented tunicamycin-stimulated upregulation of HtrA1 protein (Figure 4A) and mRNA (Figure 4B).

In metazoans, UPR relieves ER stress in two phases: a rapid and transient non-selective reduction in nascent peptide synthesis, and a more gradual, prolonged change of global gene expression (Ron and Walter, 2007). We were interested in the effect of HtrA1 ablation in this latter phase of UPR. We extracted total RNA from vehicle or tunicamycin-treated RPE cells at the 12 h timepoint for comparative gene expression analyses. In a representative subset of validated UPR genes, our treatment yielded robust response, and we observed statistically significant changes (upregulation or downregulation) in ~71% of analytes. Whereas HtrA1 knockdown under resting conditions was not deleterious to RPE, upon proteotoxicity, it showed system-wide perturbation in UPR genes (Figure 4C). In ~65% of the targets, gene expression in the face of ER stress after HtrA1 ablation was markedly different from control, with some genes changing in the opposite direction relative to control (Figure 4D).

We evaluated signal relay of the BiP-dependent effectors by treating ARPE-19 cells with a constant dose of tunicamycin and collecting total cell lysates at various time points (Figure 4E). Immunoblot analysis showed that control RPE induced early and sustained IRE1α protein expression. This correlated with auto-activation and signal transduction, as indicated by the accumulation of phospho-IRE1α and sXBP-1 within 3 h, consistent with previously reported early-onset IRE1 signaling in the adaptive phase of UPR (Calfon et al., 2002; Lin et al., 2007). In contrast, IRE1α upregulation in HtrA1 knockdown cells was delayed and correlated with relative latency and reduction in the levels of phospho-IRE1α and sXBP-1.

In control cells, PERK was downregulated after prolonged tunicamycin exposure. HtrA1 silencing resulted in sustained PERK levels and activity at all time points, as indicated by Ser51 phosphorylation of eukaryotic initiation factor 2α (eIF2α). Sustained levels of phosphoSer51 eIF2α (peIF2α) prolongs translational repression (Moreno et al., 2012) and implies an inability of HtrA1 knockdown cells to recover from ER stress. The protein expression of the pro-apoptotic transcription factor CHOP was induced after 1 h tunicamycin exposure; but unlike control cells, CHOP did not return to baseline upon HtrA1 silencing.

Finally, in control cells, two ATF6 targets cAMP response element binding protein H (CrebH), which is encoded by cAMP response element binding protein 3-like 3 (*CREB3L3*), and serum response element binding factor 1 (SREBF1) were not robustly induced until after prolonged ER stress. In contrast, HtrA1 knockdown cells had early and sustained induction of ATF6 targets. The early onset of ATF6 activation upon proteotoxicity is consistent with our observation that its functional antagonism by the IRE1 pathway via competition for unspliced XBP-1 is relieved upon HtrA1 silencing (Calfon et al., 2002; Harding et al., 2003).

We cross-validated these protein expression studies with gene expression analyses (Figure 4C and D) and grouped together the transcripts that encode signaling effectors of the IRE1, PERK, and ATF6 pathways. In HtrA1 knockdown cells, the IRE1α transcript ER-to-nucleus signaling protein 1 (*ERN1*) was not different from that in non-silencing control cells, but *ERN2* encoding IRE1β was markedly reduced. The BiP transcript heat-shock protein family A member 5 (*HspA5*) was enhanced with HtrA1 knockdown (Figure 4F). Transcripts encoding PERK (eukaryotic initiation factor 2α kinase 3, *EIF2AK3*) or eIF2α (*EIF2A*) were not differentially expressed after HtrA1 knockdown. We detected significant changes to downstream targets of PERK. *DDIT3* transcript was increased, and there was a downward trend in the transcript levels of TRIB3. Interestingly, protein phosphatase 1 subunit 15 A (*PPP1R15A*), which encodes the protein GADD34, was reduced (Figure 4G). GADD34 is a binding partner of protein phosphatase 1 (Novoa et al., 2003), which deactivates peIF2α. This supports our observation that HtrA1 knockdown results in prolonged eIF2α activation in the face of chronic proteotoxicity (Figure 4E). Finally, HtrA1 silencing enhanced *ATF6B* expression, and, with chemical ER stress induction, robustly increased transcript levels of *CREB3L3* and *SREBF1* (Figure 4H). Notably, signal transduction of ATF6 is regulated by proteolytic activation by Site 1 and/or Site 2 proteases (S1P and/or S2P), which are encoded by *MBTPS1* and *MBTPS2* (Ron and Walter, 2007). Although we observed transcriptional and translational increase in *CREB3L3* and *SREBF1* upon HtrA1 knockdown, ER stress did not increase *MBTPS1* and *MBTPS2* transcripts.

**Figure 5:**
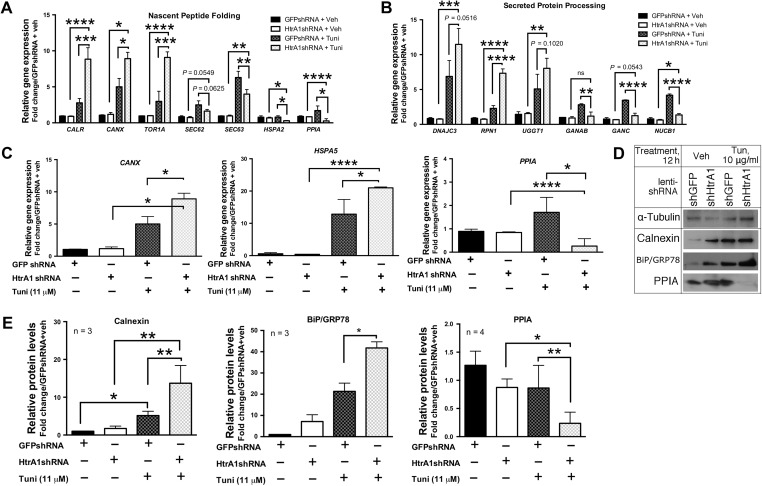
HtrA1 knockdown results in dysregulation of the chaperone network in RPE. (**A** and **B**) Gene expression analyses of control and HtrA1-deficient cells after 12 h treatment with vehicle or tunicamycin (5.62 μM) showed significant differences in chaperones involved in nascent peptide folding (**A**) or secreted protein processing (**B**). (**C**−**E**) Randomly selected analytes were validated for qRT-PCR and immunoblot consistency.

Our results indicate that suppression of HtrA1 after prolonged ER stress resulted in *en masse* transcriptional and translational perturbations that were deleterious to RPE, including widespread compensation in nascent peptide folding and secretory protein processing in the ER. Of the 31 chaperones in our probe set, 12 were significantly different between GFP shRNA and HtrA1 shRNA cells. For instance, Calreticulin and Calnexin were upregulated upon HtrA1 knockdown. The ER protein export channel Sec63 and peptidylprolyl isomerase A (PPIA) were reduced (Figure 5).

**Figure 6:**
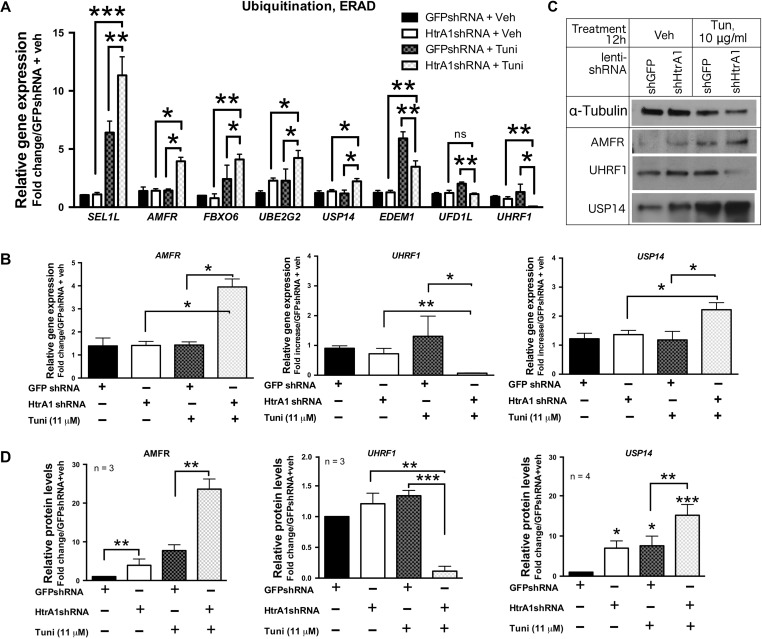
HtrA1 knockdown leads to dysregulation of the ubiquitin−proteasome system in RPE. (**A**) Gene expression analyses of control and HtrA1-deficient cells after 12 h treatment with vehicle or tunicamycin (5.62 μM) showed significant differences in genes involved in the ubiquitin-proteasome system. (**B**−**D**) Three analytes were randomly picked to validate qRT-PCR and immunoblot consistency.

Importantly, this was accompanied by dysregulation of the ubiquitin−proteasome system (Figure 6). Of the 10 ubiquitin−proteasome-related transcripts in our microarray probe set, eight were significantly different between control and HtrA1 shRNA cells. HtrA1 knockdown resulted in significant increase of the 26S-proteasome deubiquitinating enzyme ubiquitin-specific peptidase 14 (USP14). This was accompanied by downregulation of the E3 ligase ubiquitin-like with PHD and ring finger domains 1 (UHRF1). Notably, in resting cells, USP14 tonically blocks ER-associated degradation by interaction with IRE1α (Nagai et al., 2009). Resting HtrA1 knockdown cells had elevated USP14, which was further enhanced upon ER stress. This is consistent with our observation that HtrA1 knockdown suppressed levels and signaling of IRE1α (Figure 4). These data imply a positive feedback loop wherein IRE1α, at least in part, increases HtrA1 transcription (Figure 2). HtrA1, in return, is required for IRE1α to meet proper amplitude, timing, and activation upon ER stress via at least two mechanisms involving competition with ATF6 for XBP-1 and tonic block by USP14.

### HtrA1 is required for RPE survival in the face of ER stress

**Figure 7:**
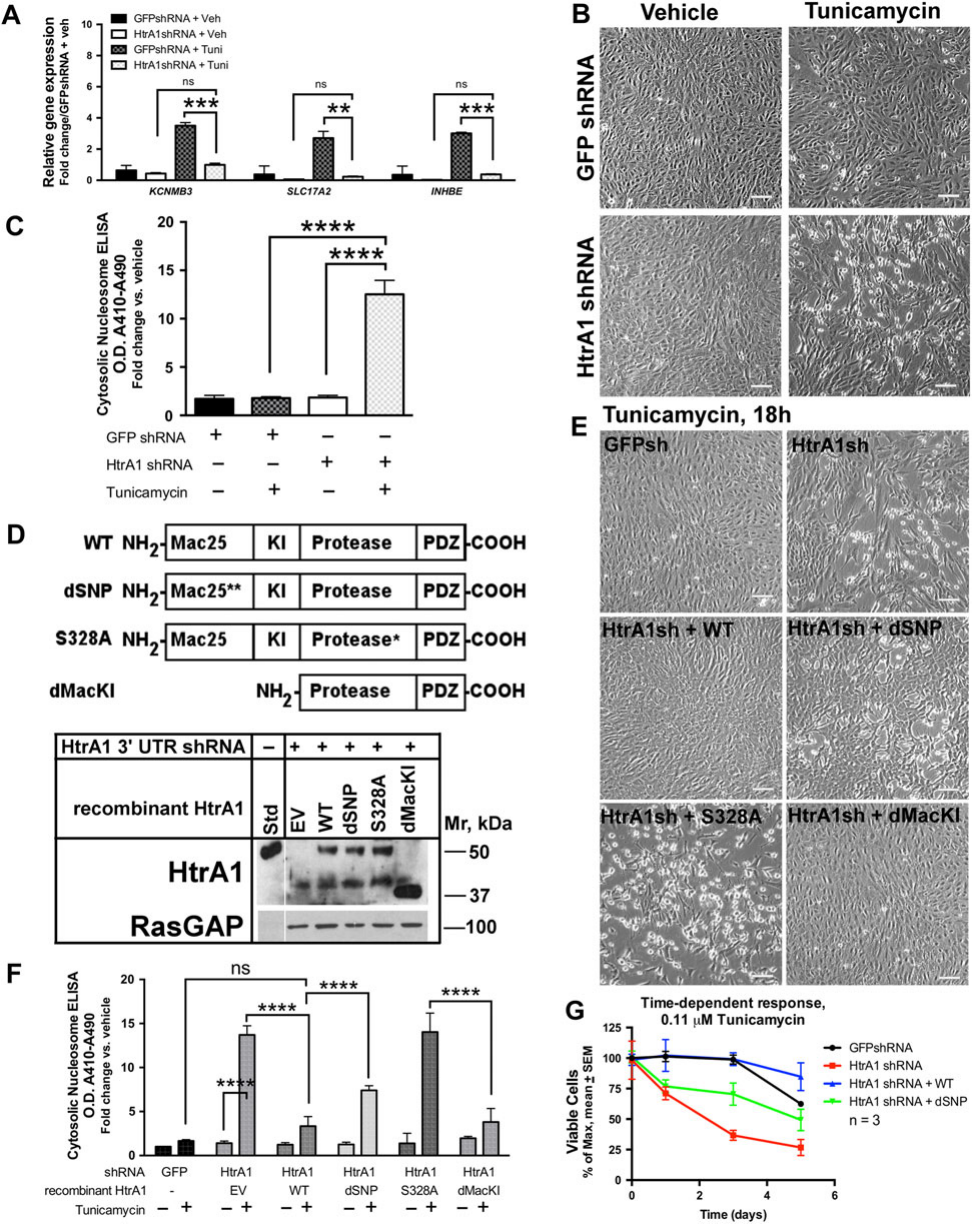
HtrA1 is required for survival in the face of proteotoxic stress. (**A**) HtrA1 silencing downregulated transcripts for indicators of cell membrane potential, including the β3 subunit of the MaxiK K+ channel (*KCNMB3*), Na^+^:PO_4_^3−^ symporter *SLC17A2*, and the growth factor modulator *INHBE.* **P < 0.01, *n* = 3. (**B**) HtrA1 silencing led to ER-associated death. Representative phase contrast images of control vs. HtrA1-deficient ARPE-19 cells after 24 h treatment with vehicle or tunicamycin (5.62 μM). Scale bar, 100 μm. (**C**) HtrA1 silencing increased ER stress-associated genomic DNA fragmentation and cytosolic nucleosome accumulation after 24 h tunicamycin (5.62 μM) treatment. ****P < 0.0001, *n* = 4. (**D**) Retrovirus-mediated gene transfer of recombinant human HtrA1 into HtrA1 knockdown ARPE-19. Immunoblot showed successful gene transfer of WT, dSNP, the protease inactive point mutant S328A, and the truncation mutant ΔMacKI into resting ARPE-19 cells expressing HtrA1 3′UTR shRNA. (**E**) Restoration of WT HtrA1 into HtrA1 knockdown RPE rescued cells from proteotoxic cell death. Serum-starved cells were treated with tunicamycin (5.62 μM) for 24 h, and brightfield images were collected to monitor cells for signs of overt cytotoxicity. Scale bar, 100 μm. (**F**) Restoration of WT HtrA1 into HtrA1 knockdown RPE suppressed stress-induced genomic DNA fragmentation. (**G**) Substitutions in HtrA1 Mac25 domain diminished ER stress threshold of RPE to subtoxic dose of tunicamycin.

We next asked whether rewiring of UPR as a result of HtrA1 ablation was a pro-survival adaptation to ER stress. Gene expression analyses revealed that cell viability indicators, such as *KCNMB3* and *SLC17A2*, which encode proteins that regulate cell membrane potential, showed differential expression after HtrA1 silencing (Figure 7A). In addition, overnight treatment with tunicamycin was cytotoxic to RPE that express HtrA1 shRNA, but not to those with non-silencing control (Figure 7B). We detected elevated levels of fragmented DNA in stressed HtrA1 knockdown cells (Figure 7C). These lines of evidence suggest that in RPE that are nonetheless able to upregulate quintessential chaperones like BiP and GRP94, HtrA1 ablation in the face of ER stress results in cell death.

We next inquired what minimum requirements within the HtrA1 protein architecture are required for RPE survival in the face of ER stress. Allelic variants of *HTRA1* mapped to the exon 1 are enriched in a subset of AMD patients (Dewan et al., 2006; Yang et al., 2006; DeAngelis et al., 2008). Of particular interest are those harboring Ala34(C > T) and Gly36(G > T) (Supplementary Figure S6A and B) within the very N-domains that were acquired by gene fusion in the vertebrate lineage. Previously, we demonstrated that the protein product harboring these silent substitutions displayed altered biophysical properties that were due, in part, to frequent-to-rare conversion of codons (Jacobo et al., 2013). Applying similar rare vs. common codon analyses (McInerney, 1998) to the full-length exon 1, we matched the codon usage for degenerate amino acids as a function of the conserved domains (Supplementary Figure S4C). The residues forming ordered α-helical or β-stranded structures were spanned by common, rapidly translated codons, whereas islands of rare, slowly translated codons correlated with intrinsically disordered loops (Komar et al., 1999). In this heat map, Ala34(C > T) and Gly36(G > T) extended a rare codon cluster, and thus likely, at least in part, accounts for the reduction in mRNA translation speed and altered protein surface accessibility for the misfolding prone ‘double single nucleotide polymorphisms’ (dSNP) HtrA1 variant (Jacobo et al., 2013). We also observed lower levels of dSNP HtrA1 compared to WT in the absence of a continuous supply of newly synthesized HtrA1 to replace those post-translationally degraded. Thus, the silent substitutions in N-domains of HtrA1 modify post-translational stability and turnover.

We tested the hypothesis that HtrA1 N-domain variants compromise adaptive UPR. We chemically induced ER stress with tunicamycin (1.12 μM, 18 h) in B-lymphocytes derived from unaffected controls and AMD affected subjects, who are homozygous for *HTRA1* variants as described above, and evaluated gene expression of UPR indicators. B-lymphocytes from unaffected controls enhanced gene expression of *XBP-1* and *TRIB3* in response to ER stress. In contrast, this was not observed in affected samples harboring homozygous HtrA1 N-domain substitutions (Supplementary Figure S6F).

Next, we delivered lentivirus-encoded shRNA, which targets a portion of the 3′ untranslated region (UTR) of HtrA1 transcript to ARPE-19. For gene replacement, knockdown cells were thereafter transduced with empty vector (EV) retrovirus or retrovirus harboring HtrA1-coding region for recombinant WT, dSNP, Ser328Ala, or truncated Mac25KI, but not 3′UTR. Immunoblot analyses of ARPE-19 total cell lysates showed successful step-wise generation of cell lines for mutational analyses (Figure 7D).

Under treatment conditions that were toxic to HtrA1 knockdown cells, restoration of WT prevented ER-associated cell death. The protease inactive S328A mutant failed to rescue knockdown cells from apoptosis. Truncation of the N-domains did not appear to severely compromise RPE survival in the face of proteotoxicity (Figure 7E and F). In contrast, RPE cells harboring dSNP showed diminished tolerance to ER stress, although the overexpression levels were comparable with WT (Figure 7F). Furthermore, restoration of WT to knockdown cells suppressed genomic DNA fragmentation in response to tunicamycin, while re-expression of dSNP showed reduced effect (Figure 7G). Indeed, RPE harboring dSNP had diminished tolerance for a subtoxic dose and prolonged exposure to tunicamycin. We conclude that although the absence of the N-domains did not appear to diminish the efficacy of HtrA1 protein to rescue knockdown cells, the presence of deleterious HtrA1 substitutions result in a protein product with reduced ability to protect RPE from ER stress-associated cell death.

## Discussion

We demonstrate in this study that HtrA1, a multi-domain protein that is frequently variant in a subset of AMD patients, performs intracellular functions in RPE. Our data show that HtrA1 plays a role in the homeostatic establishment of protein quality control and is essential for RPE survival under conditions of proteotoxicity. Deficits in HtrA1, mimicked here by suppressed availability of exported or intracellular pools, loss in serine protease activity, or the presence of a misfolding-prone N-terminal moiety, compromised tolerance to proteoxicity, in spite of retaining ability to induce quintessential chaperones.

The historic link between *HTRA1* genetic variants and inherited susceptibility to AMD have been called into question by more recent association studies with increased population sizes and refined statistical models (Grassmann et al., 2017). In addition, although various overexpression studies in the mouse eye have implicated HtrA1 in the proteolysis of Bruch’s membrane’s and enhanced choroidal neovessel extravasation (Jones et al., 2011; Vierkotten et al., 2011), these phenotypes are poorly penetrant in mice even after achieving double-digit augmentation or combination with environmental risk factors (Kumar et al., 2014; Nakayama et al., 2014). Taken together with our findings, a paradigm that emerges suggests that although HtrA1 may not be a major culprit in inherited risk to AMD, it appears to have rudimentary functions in stress response.

In our gene replacement experiments, the protective function of HtrA1 against proteotoxicity is compromised by a conformationally altered N-terminal moiety. Previous studies highlighted the frequency of intrinsically disordered regions in gained domains of multi-domain proteins (Buljan et al., 2010). It is tempting to speculate that our data hints at the origins of intrinsic disorder from ancestral noncoding sequences that eventually became exonized. This raises the interesting possibility of altered assembly of quaternary complex, modified substrate-induced activation, subcellular localization, and importantly, binding partners that may have emerged upon N-domain accretion.

We traced the evolutionary origins of N-domains in HtrA family to the acquisition of a single exon from one of the ancestral IGFBP-like proteins (IGFB7) in *Ciona*. In a previous report, when HtrA1 far exceeds IGF-1 levels in cell-free solution, we co-immunoprecipitated IGF-1 with purified, full-length or affinity-tagged Mac25/KI fragments of HtrA1, and pre-incubation of IGF-1 with HtrA1 prevented ligand-mediated IGF1R activation in cultured endothelial cells (Jacobo et al., 2013). In our affinity measurements by ELISA and titration immunoblot, the affinity of HtrA1 for IGF-1 was 10 μμM and several orders of magnitude above that of IGFBPs (1 pM) or IGF-1R (10 pM) (Rodgers et al., 2008). We were able to reproduce TGFβ1 co-immunoprecipitation with HtrA1 as previously reported (Oka et al., 2004). These data point to the conclusion that although N-domains of human HtrA1 retained the structure of homologous conserved domains, they have lost stereotypical functions of those discrete units. Interestingly, vasculopathy-associated mutations in HtrA1 that abolish its protease activity (Hara et al., 2009) and ability to oligomerize (Nozaki et al., 2016), but harbor the anti-TGFβ-like KI domain, are unable to antagonize TGFβ signaling. The *in vivo* relevance of human HtrAs serving as a growth factor sink is unclear, partly because there is a circulating suicide inhibitor in the blood (Hu et al., 1998), and only HtrA1 mutants that lack serine protease activity (Hara et al., 2009) or ability to oligomerize were able to evade covalent complex formation with α1-antitrypsin (Nozaki et al., 2016).

Sequestration of exported HtrA1 with neutralizing antibodies did not compromise RPE survival in the face of ER stress. Indeed, most of the HtrA1 we detected in ARPE-19 was intracellular, though ER stress did not prevent exit traffic from cells. These findings raise the question how cells export HtrA1. HtrA1 has been detected in its full-length precursor form with the signal peptide still intact (Didangelos et al., 2010) in exosomes derived from RPE (Wang et al., 2009), heart (Prunotto et al., 2013), and osteoblasts (Xiao et al., 2007). These antecedents, and observations that HtrA1 lacks glycosylation (Yin et al., 2013), prompted us to visualize subcellular localization of HtrA1. We found that HtrA1 fully co-aligned with vimentin IFs. Co-localization with MTs was only partial, and specifically, detectable where there is Ac-α-tubulin. Thus, apart from the regulation of cell cycle (Schmidt et al., 2016) and migration (Chien et al., 2009), HtrA1 association with these cytoskeletal filaments is critical to rudimentary proteome maintenance.

In summary, our findings unravel a possible role for HtrA1-mediated RPE damage in AMD. Deleterious substitutions in HtrA1 lower the ER stress threshold of RPE, and collapse in proteome homeostasis may be an event that contributes to RPE degeneration in the subset of patients with genetic lesions in HtrA1. The subcellular localization of HtrA1, mode of extracellular export, and repertoire of binding partners illuminate design requirements for interventional strategies that aim to target HtrA1 and HtrA1-mediated signaling pathways for therapy.

## Materials and methods

### Phylogenetic analyses

HtrA protein sequences were compiled from a diverse set of species. Most sequences came from Ensembl (Yates et al., 2016), except those from *Branchiostoma floridae* (Putnam et al., 2008) and *Callorhinchus milli* (Venkatesh et al., 2014). One of the sequences from *Petromyzon marinus* (ENSPMAP00000002693) had an N-terminal KI domain, but not Mac25 domain. However, upon inspection of the Ensembl Genscan *ab initio* predicted proteins, a further N-terminal extension of the sequence was identified that included the Mac25 domain, and the sequence was extended to include this. Another *Petromyzon marinus* sequence (ENSPMAP00000002696) did not have Mac25 or KI domains, but inspection of the *ab initio* sequences revealed an N-terminal extension containing a KI but not Mac25 domain. All sequences are provided in Supplementary Dataset S1. Domain assignments were performed for all sequences using SUPERFAMILY (Oates et al., 2015). Domains labeled as Mac25, KI, serine protease, and PDZ were predicted to belong to the ‘growth factor receptor domain’, ‘Kazal-type serine protease inhibitor’, ‘trypsin-like serine protease’, and ‘PDZ domain-like’ families, respectively.

A multiple sequence alignment was generated using ProbCons (Do et al., 2005). This was used with MrBayes (Ronquist et al., 2012) to generate a phylogenetic tree, using a mixed amino acid model and a 4-category gamma distribution with invariant sites. Using Markov Chain Monte Carlo, two chains were run for 1.2 × 10^6^ generations with sampling every 100 generations. The average standard deviation of split frequencies was 0.005. The phylogenetic tree was visualized and midpoint rooted using FigTree. Domain schematics were generated with PROSITE MyDomains (Hulo et al., 2008).

### Tunicamycin-induced proteotoxicity in ARPE-19

Reagents used in this study were from Sigma-Aldrich unless otherwise indicated. ARPE-19 (ATCC) were seeded in 12-well plates (250 × 10^3^ cells/well) using DMEM/F12 with 10% FBS. Cells were serum-starved for 24 h prior to the experiment, and then treated with tunicamycin or vehicle (DMSO) at various time points. Cells were washed with PBS and lysates were harvested for real-time qRT-PCR or immunoblot for markers of UPR. In Figure 2C and D, we pre-incubated ARPE-19 cells for 1 h with the IRE1α inhibitor 4μ8C (Tocris Bioscience; 7-Hydroxy-4-methyl-2-oxo-2*H*-1-benzopyran-8-carboxaldehyde). Alternatively, we also treated cells with a kinase inhibitor to PERK, GSK2606414 (Tocris Bioscience, 1-[5-(4-Amino-7-methyl-7*H*-pyrrolo[2,3-*d*]pyrimidin-5-yl)-2,3-dihydro-1*H*-indol-1-yl]-2-[3-(trifluoromethyl)phenyl]ethanone). For side by side comparisons of the efficacy of oxidant stressors in inducing HtrA1 expression, ARPE-19 cells were cultured and serum-starved as described above, and thereafter exposed to H_2_O_2_ (0.5 mM) or nicotine (2.0 μM) for 24 h. Total cell lysates were collected for immunoblot. For measurements of cell viability (Figure 7), cells were treated with tunicamycin and monitored for signs of cell death under brightfield microscopy, and viable cells were counted with hemocytometer.

### Real-time qRT-PCR

For all real-time qRT-PCR analyses, ARPE-19 cells were seeded in 6-well plates (500 × 10^3^ cells/well). Cells were washed 2× with PBS, and then lysed in RNA-bee (AMS Bio) for total RNA collection. For one-step qRT-PCR in Figure 1B, 100 ng total RNA was used as template in a 20 μl reaction using a Power SYBR Green RNA-to-C_T_ 1-step kit (Applied Biosystems). For all other qRT-PCR experiments, 1.0 μg total RNA was used in a 20 μl reverse transcription reaction using a High-Capacity cDNA Reverse Transcription Kit. This RT reaction was diluted 10-fold, and 2 μl was used in a 20 μl real time PCR with Power SYBR Green PCR master mix (Applied Biosystems). The probes used in these studies are listed in Supplementary Table S2. In addition, we collected total RNA after 12 h of tunicamycin exposure for the RT^2^ Profiler^TM^ PCR Array Human Unfolded Protein Response (Qiagen). Data QC and analyses were performed as described by Qiagen.

### Western blot

Total cell lysates (50 μg/well) were resolved in 4%−16% tris-glycine gels (Bio-Rad) and transferred to PVDF membranes. For comparisons of relative secreted vs. intracellular HtrA1, 24 h conditioned media were collected and clarified of cell debris by centrifugation for 1 min at 13000*× g*. An aliquot of conditioned media (50 μl of 1000 μl in total) or total cell lysates (50 μg of ~1000 μg in total) were resolved in protein gels side by side with known quantities of purified full-length human HtrA1 standard (B-Bridge International). Membranes were incubated overnight with primary and HRP-conjugated secondary antibodies (Supplementary Table S3) and developed with ECL.

### Immunofluorescence staining

ARPE-19 cells were cultured to confluence on laminin-coated glass coverslips. Cells were serum-starved for 24 h, rinsed, and treated for 6 h with vehicle (DMSO) or tunicamycin (5.62 μM) in serum-free DMEM/F12. In Figure 3, after 4 h of vehicle or tunicamycin treatment, nocodazole (5 μM) was added to the culture medium and cells were exposed to the treatments for an additional 2 h. At the end of the assay, cells were loaded with 1.0 μM ER tracker DPX blue dye (Molecular Probes, ThermoFisher) and incubated in the dark for 15 min at 37°C. Cells were rinsed with PBS and immediately fixed in 4% PFA for 5 min at RT. Cells were permeabilized and blocked for non-specific immunoreactivity with buffer containing 0.1% Triton X-100, 3% BSA, and normal serum (5% final, Sigma-Aldrich). Cells were stained overnight with HtrA1 antibodies and co-stained with cytoskeletal (α-tubulin or vimentin) or ER markers (Calreticulin). For negative control, an equal molar dose of non-immune rabbit IgG (sc-2027, Santa Cruz Biotechnology) was used in place of the anti-HtrA1 antibody. Cells were rinsed and incubated with Alexa Fluor 488-conjugated (for mouse anti-HtrA1) or Alexa Fluor 594-conjugated (for counterstained protein markers) AffiniPure goat anti-rabbit F(ab)2 (Jackson Immunoresearch) for 1 h at RT. Coverslips were mounted on Ultra^TM^ Frost glass slides (Denville Scientific) with Prolong Gold antifade reagent (Cell Signaling Technology).

### Laser-scanning confocal imaging

All imaging experiments are representative of four independent experiments with duplicate coverslips. For each coverslip, randomly selected fields were imaged by sequential scanning of each channel using a Leica upright DM 6000S. RGB images at 630× magnification were acquired from optical slices of <3.0 μm thickness at a resolution of 600 ppi using Confocal Leica Application Suite AF software (v. 1.3.1). Images were imported as 16-bit grayscale images into Fiji ImageJ. In every field, ~10−15 cells were highlighted as ROI and analyzed using Coloc2 and Colocalization Threshold. The pixel intensity correlation of HtrA1 and counterstain were expressed as Pearson’s correlation coefficient and co-localized pixel maps. The differences in proportion and co-occurrence in objects from different channels were accounted for Manders correlation coefficient with Costes thresholding and plotted fractional channel overlap as percentage.

### Lentivirus-mediated HtrA1 knockdown

Short hairpin RNAs that target human HtrA1 exons were selected from The RNAi Consortium of Broad Institute and synthesized at the Core-RNA Interference Screening Facility of Dana-Farber Cancer Institute. We selected the pLKO.1-encoded shRNA with the target sequence 5′-CGCCATCATCAACTATGGAAA-3′. Viral particles containing the shRNAs were produced by triple transfection of HEK293T cells (1.0 × 10^6^) with 1.0 μg total cDNA using TransIT293 (Mirus Bio). The target plasmid was mixed with pCMV-ΔR8.91 packaging and pMD2.G envelope plasmids to produce VSV-G pseudotyped lentiviral particles that were used to infect P20-21 ARPE-19. Stable cell lines harboring this exon-targeted HtrA1 shRNA were used for Figure 4 and Supplementary Figures S3 and S4. For gene replacement experiments, we generated and screened three hairpin sequences that target different portions of the 3′UTR of the human HtrA1 mRNA, and selected the forward oligonucleotide 5′-CCGGAGAATCCTTCTTGATAGTTTGCTCGAGCAAACTATCAAGAAGGATTCTTTTTTG-3′ and the reverse oligonucleotide 5′-AATTCAAAAAAGAATCCTTCTTGATAGTTTGCTCGAGCAAACTATCAAGAAGGATTCT-3′. These were annealed, ligated to pLKO.1, and validated by Sanger Sequencing (MGH DNA Sequencing Core). Lentiviral particles were generated for infecting ARPE-19 cells. Stable cell lines harboring this 3′UTR-targeted HtrA1 shRNA plus recombinant cDNA were used in Figure 7. All experiments described here used cells that were selected in puromycin (2.0 μg/ml) and thereafter validated for HtrA1 knockdown using western blot and qPCR.

### Ethics statement

This study was reviewed and approved by the Institutional Review Boards at the Massachusetts Eye and Ear Infirmary and the University of Utah, and conforms to the tenets of the Declaration of Helsinki. Written informed consent was obtained from all participants.

### Subjects and phenotypes

Details of recruitment diagnostic criteria and subject classification were described previously (Silveira et al., 2010). Genotype and statistical analyses of human subject data were described in detail previously (Jacobo et al., 2013). Data in Supplementary Figure S4A, B and Table S1 differ from this prior work in the inclusion of early AMD and geographic atrophy (GA) patients.

### Post-translational degradation of HtrA1

We transfected 1.0 × 10^6^ HEK293T cells with 1.0 μg plasmid encoding EV (pCINeo, Promega), WT, or dSNP. At 24 h post-transfection, cells were washed and serum-starved in DMEM/F12 for 24 h. At the peak of protein expression (48 h), transfected cells were treated with cycloheximide (CHX, 100 μM) to inhibit nascent protein synthesis. After 8 h, total cell lysates were collected and analyzed by immunoblot. The amount of HtrA1 in the CHX-treated cells reflects the net turnover at the peak of transfection minus that from post-translational degradation, in the absence of new HtrA1 synthesis.

### Retrovirus-mediated expression of recombinant HtrA1

Recombinant human HtrA1 cDNA templates were PCR-amplified using the forward primer 5′-CATGCGGCCGCCACCATGCAGATCCCGCGCGCCGCT-3′ and the reverse primer 5′-AATGTCGACCTATGGGTCAATTTCTTCGGGAATCAC-3′ from pCINeo templates encoding wild-type (WT), ‘double SNP’ (dSNP), S328A, and truncated HtrA1 that lacks Mac25 and KI domains (dMacKI) as previously described (Jacobo et al., 2013), and then cloned into the *Not*I and *Sal*I sites of the retrovirus vector pLNCX2. All cDNA plasmids were validated by Sanger Sequencing. The target plasmids were packaged into VSV-G pseudotyped retrovirus, and then used for transducing puromycin-resistant ARPE-19 cells that stably express HtrA1 mRNA 3′UTR-specific shRNA (described above). Retrovirus-transduced ARPE-19 were selected using G418 (1.2 mg/ml), and neomycin-resistant ARPE-19 were validated for recombinant HtrA1 overexpression using immunoblot.

### Cytosolic nucleosome ELISA

Briefly, 96-well clear-bottomed plates were coated with anti-histone antibody for 1 h at RT. ARPE-19 total cell lysates (5 μg/well) were diluted in the incubation buffer to a final volume of 100 μl/well. Samples in duplicate were equilibrated on the coated plates for 90 min at RT. To measure apoptosis-associated DNA fragmentation, we followed the manufacturer’s protocol for Cell Death ELISA (Roche Life Sciences).

### Data analysis and statistics

Data analyses were performed with Prism 4.0 (GraphPad). Where appropriate, two-tailed student’s *t*-test was used for paired comparisons. One-way ANOVA followed by Tukey’s *post-hoc* test was used for comparison of more than two groups; Dunnett’s *post-hoc* test was used for comparison of multiple groups against vehicle or time 0 baseline. Comparisons wherein *P < 0.05 at α ≥ 0.05 were considered statistically significant.

## Supplementary material

Supplementary material is available at *Journal of Molecular Cell Biology* online.

## Funding

S.M.P.J. was supported by a Macular Degeneration Grant from BrightFocus Foundation (M2014025). M.J.G. and S.R. were supported by the Koeln Fortune Program Award (KF Nr. 249/2013) from the Faculty of Medicine, University of Cologne, Germany. J.A.M. was supported by a Medical Research Council Career Development Award (MR/M02122X/1). M.S.-G. was supported by the U.S. National Institutes of Health grant 1R01EY023682, the Grimshaw Foundation, the Research to Prevent Blindness Dolly Green Special Scholar Award, and donations to the Macular Degeneration Research, a program of the BrightFocus Foundation (M2013161). M.M.D. was supported by an unrestricted grant from Research to Prevent Blindness Inc. to the Department of Ophthalmology and Visual Sciences, University of Utah, the U.S. National Institutes of Health core grant EY014800, Carl Marshall Reeves & Mildred Almen Reeves Foundation, Inc., and Macular Degeneration Foundation. All confocal imaging experiments at the Schepens Microscopy Core Facility were supported by the U.S. National Institutes of Health National Eye Institute core grant P30EY003790.

**Conflict of interest:** one of the SNPs is included in Patent No. US 7,972,787, B2 to M.M.D. and MEEI.

**Author contributions:** S.M.P.J. conceived the study and performed biochemical experiments. M.J.G. generated lenti/retrovirus particles, constructed stable cell lines, and performed experiments with S.M.P.J. S.M.P.J. and J.A.M. performed phylogenetic analyses. cDNA constructs, viral vectors, and cell lines using these reagents were designed and generated by S.M.P.J. in Kazlauskas Lab. Ar.K. assisted with confocal imaging. M.M.D. and M.M. genotyped patients, performed all genetic studies, and generated B-lymphocyte cell lines from normal and AMD subjects. S.M.P.J., M.J.G., J.A.M., M.M., S.R., M.M.D., and M.S.-G. analyzed data. M.J.G., J.A.M., and S.M.P.J. wrote the manuscript.

## Supplementary Material

Supplementary Data
