# Supplementary material for: ER stress-induced aggresome trafficking of HtrA1 protects against proteotoxicity
Source: J Mol Cell Biol. 2017 Aug 10;9(6):516–32. doi: 10.1093/jmcb/mjx024 (PMC5823240; doi:10.1093/jmcb/mjx024)
Supplement: Supplementary Data [file mjx024_supplemantarymaterial.pdf]

Supplementary Figures

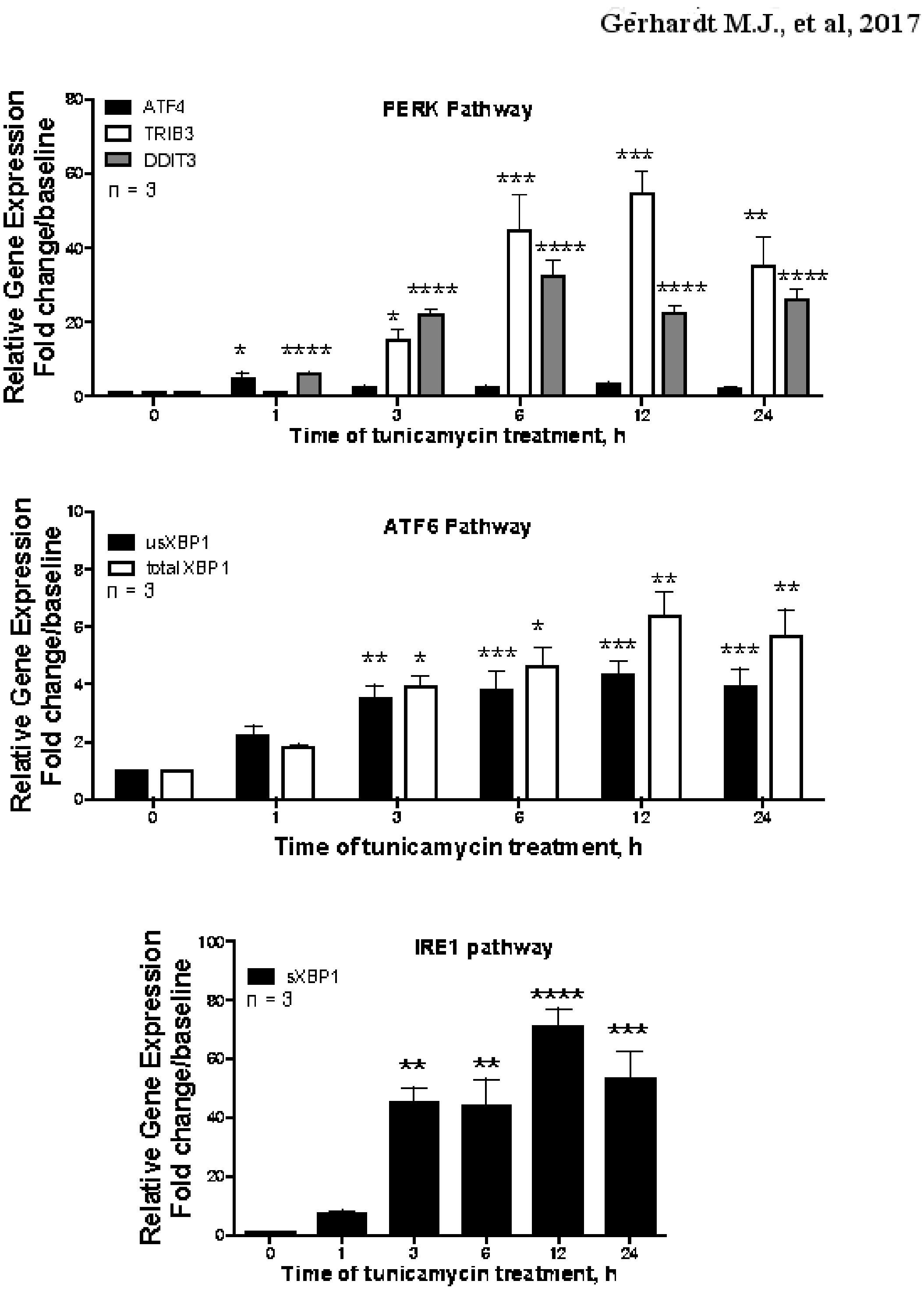

Supplemental Figure 1

Supplementary Figure S1. Unfolded protein response mediators in RPE after chemical induction of ER stress. To evaluate whether the three branches of BiP-mediated UPR were in place and active in RPE after ER stress induction with tunicamycin (5.62 μM), we collected total RNA at the indicated time points for qRT-PCR analyses. We validated that RPE is able to activate signal transduction downstream of PERK (A), ATF6 (B) and IRE1α (C). The PERK targets *ATF4* and *DDIT3* were up-regulated as of 1h of tunicamycin exposure. *DDIT3* transcriptional up-regulation was sustained, but *ATF4* eventually returned to baseline after prolonged chemical ER stress induction. ATF6 and IRE1α targets were upregulated after 3h, and remained high up to 24h.

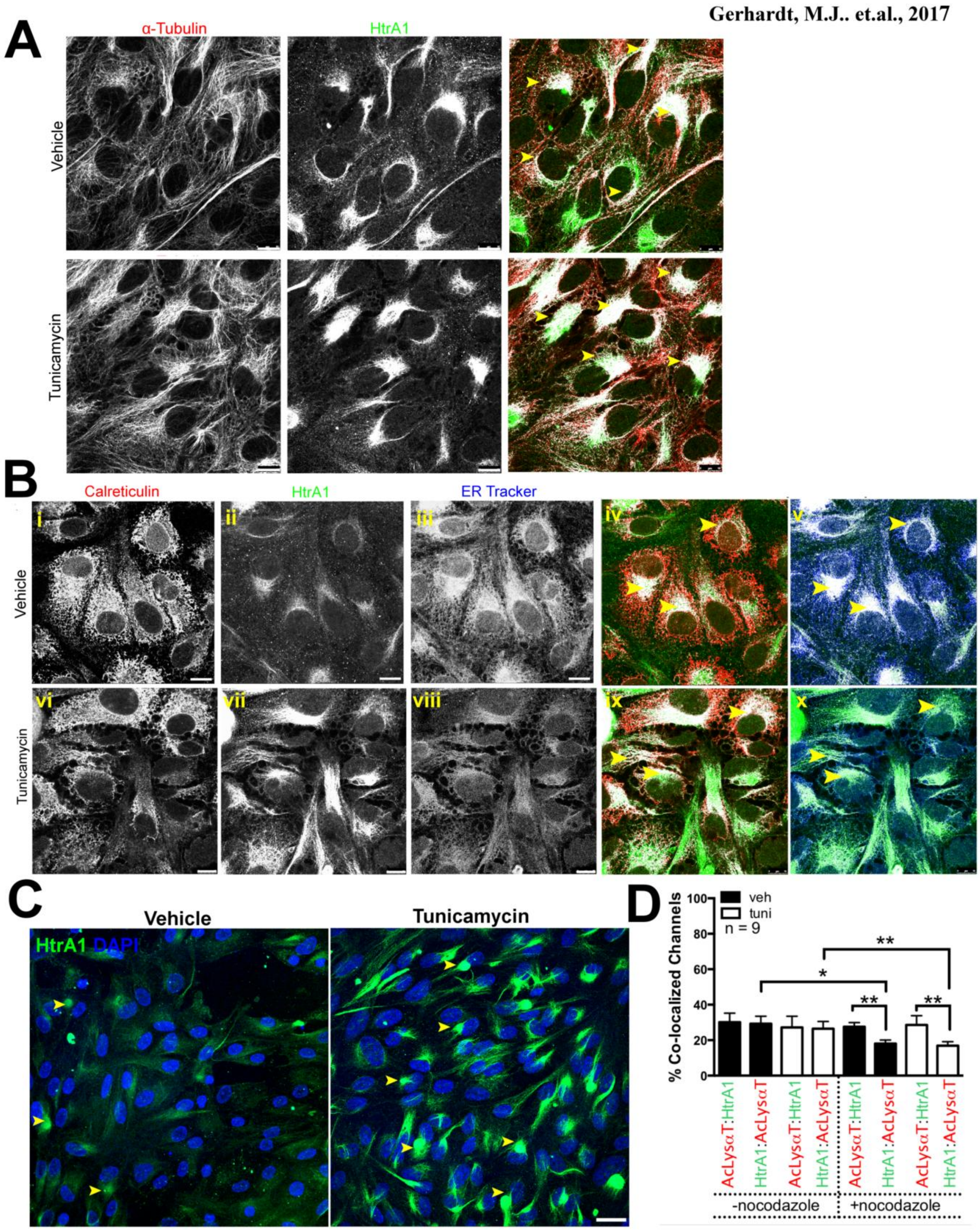

Supplemental Figure 2

Supplementary Figure S2. HtrA1 associates with the cytoskeleton and partially overlaps with ER. A. HtrA1 (middle panels) partially overlaps with microtubules (left panels) at the microtubule organizing center (MTOC). Co-localized pixel maps (right panels) were generated with Fiji. Yellow arrowheads show examples of cells wherein HtrA1 overlapped with α-tubulin at the MTOC. Scale bar = 10 μm.

B. HtrA1 partially overlaps with Calreticulin and ER Tracker. In vehicle-treated cells, HtrA1 immunostaining appeared as arborized structures that partially overlapped with the ER protein and lipid markers Calreticulin and ER tracker around the nucleus (panels i - iii). In tunicamycin-treated cells, HtrA1 was enriched in flattened, stacked, sheet-like structures that were enriched at pericentriolar foci (panels iv – vi). Scale bar = 10 μm.

C. Representative wide-field images of HtrA1 showing up-regulation and enrichment at collapsed, sheet-like structures at the MTOC after ER stress induction with 6h tunicamycin treatment. Scale bar = 100 μm.

D. De-stabilization of microtubules with nocodazole partially uncouples HtrA1 from acetylated MT. Fractional red:green and green:red channel overlap shows that nocodazole treatment reduced HtrA1 co-distribution with nocodazole-resistant acetylated α-tubulin. Cells were manually selected as ROI per field, and we quantified co-localization as described in Figure 3. In one-way ANOVA with Tukey's post-hoc test, $^*p<0.05$.

Gerhardt, M.J., et. al., 2017

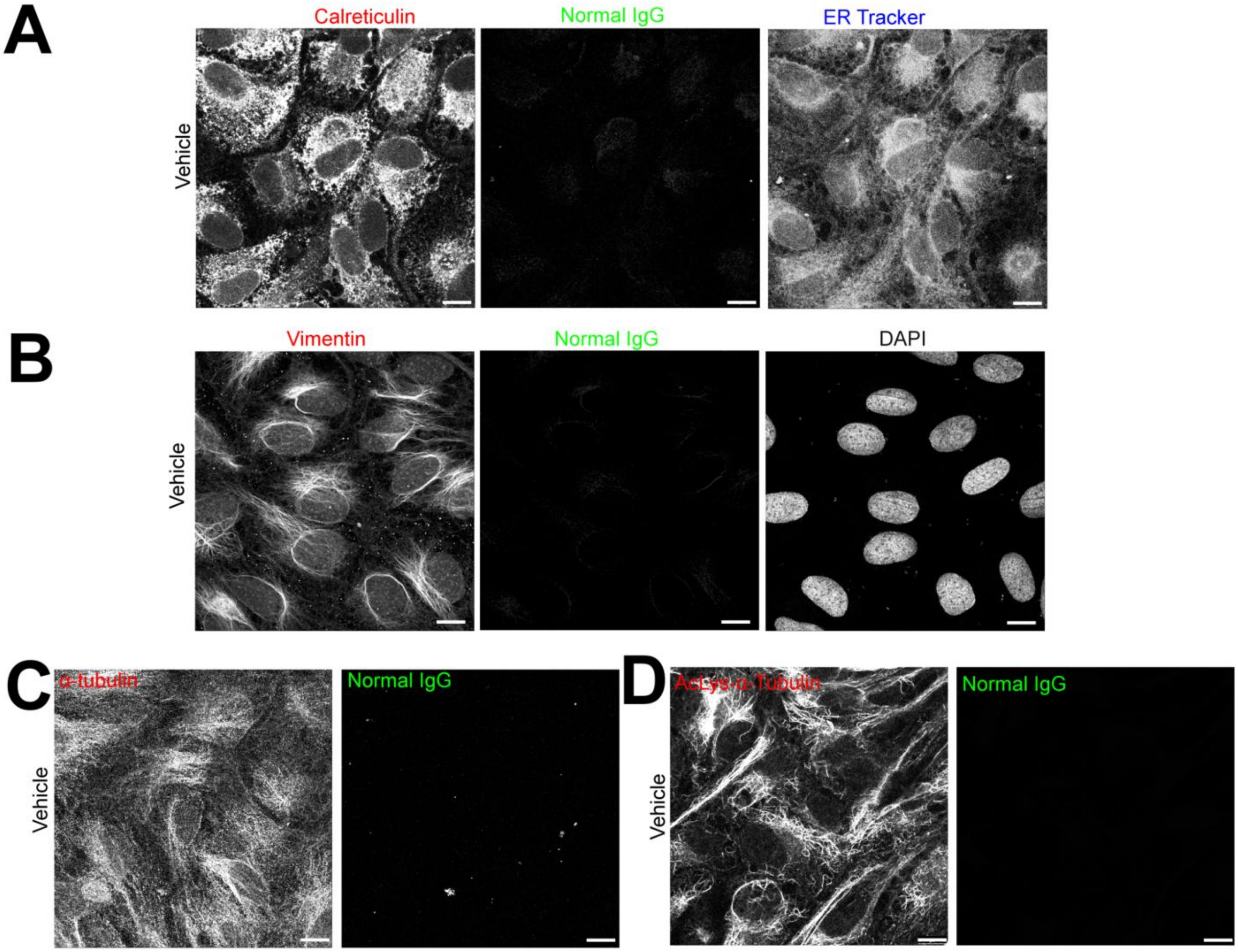

Supplemental Figure 3

Supplementary Figure S3. Localization of HtrA1 is dependent on the cytoskeleton.

A. Negative control for HtrA1 immunostaining when ER is counter-stained with Calreticulin and ER tracker.

B. Negative control for HtrA1 immunostaining when intermediate filaments are counter-stained with vimentin.

C. Negative control for HtrA1 immunostaining when microtubules are counter-stained with α-tubulin.

D. Negative control for HtrA1 immunostaining when microtubules are counter-stained with AcLys40-α-tubulin.

Gerhardt, M.J., et. al. 2017

A

HTRA1		Normal		Dry AMD		Geographic Atrophy		Neovascular AMD	
		Frequency %	No.	Frequency %	No.	Frequency %	No.	Frequency %	No.
rs1049331 + rs2293870	Genotypes								
	Homozygous risk at both	13.92%	27	26.53%	26	66.67%	8	33.73%	112
	At least 1 risk allele at both	48.45%	94	55.10%	54	83.33%	10	71.39%	237
	Total		121		80		18		349

B

		All AMD (Neo, Dry, GA) vs. Normal			
SNP	Allele	Odds Ratio	95% CI Low	95% CI High	p value
rs2293870add	T or C	3.497	2.224	5.499	5.94E-08
rs2293870dom	T or C	4.199	2.154	8.186	2.53E-05
rs2293870rec	T or C	4.987	2.517	9.881	4.11E-06
rs1049331add	T	5.676	3.280	9.823	5.46E-10
rs1049331dom	T	6.044	3.046	11.990	2.64E-07
rs1049331rec	T	9.720	3.730	25.331	3.26E-06

C

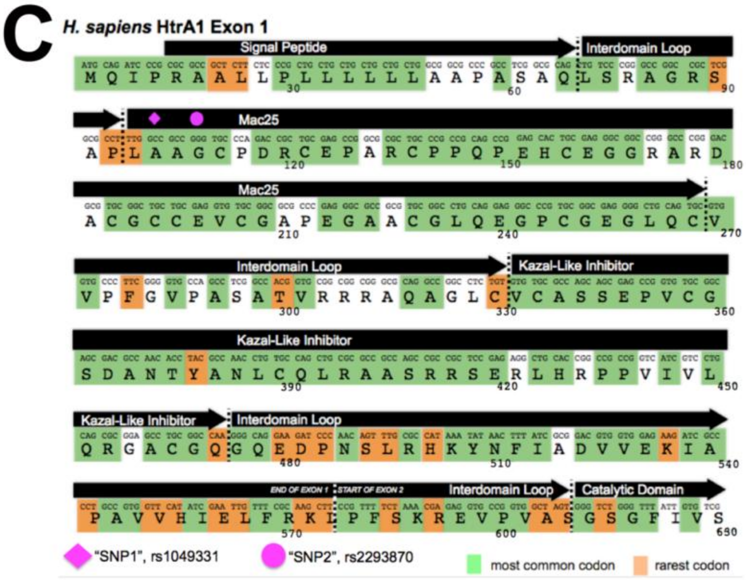

D

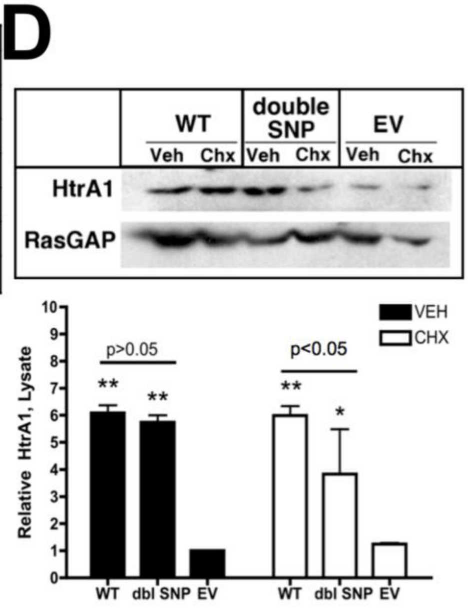

E

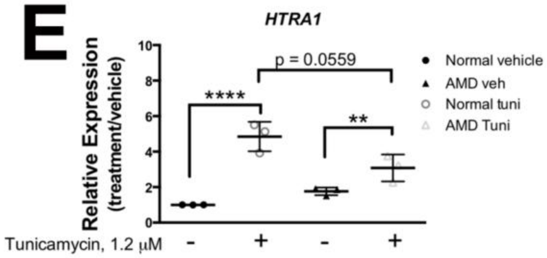

F

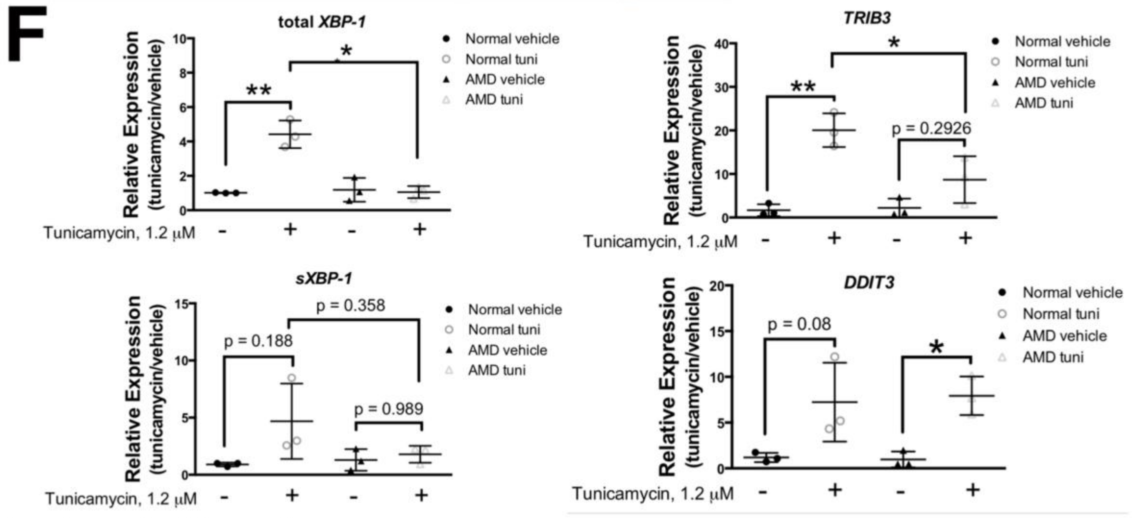

Supplemental Figure 4

Supplementary Figure S4: Silent substitutions in *HTRA1* alter UPR genes in patient-derived cell lines and predict diminished ER stress capacity

A. Synonymous substitutions rs1049331 and rs2293870 in *HTRA1* are enriched in representative populations of subjects with dry AMD, geographic atrophy, or neovascular AMD, relative to normal control subjects. Minor allele frequencies for individuals who are homozygous at both or heterozygous for at least one of the two alleles are tabulated.

B. Synonymous substitutions rs1049331 and rs2293870 in *HTRA1* increase the risk (odds ratio) of developing AMD.

C. Synonymous substitutions rs1049331 and rs2293870 in *HTRA1* extend a rare codon cluster. Each degenerate amino acid in the first exon of the HtrA1 transcript was scored green if encoded by the most common codon, or yellow if encoded by the rarest codon, using the human table from the Graphical Codon Usage Analyzer (http://gcua.schoedl.de) (McInerney, 1998). Amino acid positions that are not scored are encoded by an intermediate-abundance codon. The linear sequence is presented as a heat map, and the conserved domains are marked with solid bold arrows above the sequence. The location of the codons for Ala34 and Gly36, which are altered by the variants rs1049331 and rs2293870 are marked with solid shapes in pink.

.

D. Synonymous substitutions in HtrA1 diminished post-translational protein lifetime in cells. We predicted that the extension of a rare codon cluster in the double SNP, "dSNP" HtrA1 transcript would compromise protein folding and stability. To test this, we transiently transfected HEK293T with cDNA that encodes WT human HtrA1 or dSNP. Empty vector was used as negative control. At the peak of protein expression (48h post-transfection), we found that steady state level of WT was comparable with that of dSNP. We eliminated the pool of newly synthesized HtrA1 by adding cycloheximide (100 μg/ml) for 8h to our cell culture before harvesting total cell lysates (56h post-transfection). The amount of HtrA1 in cycloheximide-treated cells indicates the pool remaining after post-translational degradation, in the absence of replenishment from nascent protein synthesis. We found that dSNP HtrA1 was significantly diminished compared to the steady-state peak levels. Immunoblots are representative of n = 3 independent experiments. In one-way ANOVA with Tukey post-hoc test, *$p<0.05$. Unless indicated by brackets, the * above the bars indicate comparison with EV-transfected controls cells that were treated by vehicle.

E. Patient-derived lymphocytes from normal (n = 3 cell lines) or AMD (n = 3 cell lines) subjects upregulate HtrA1 transcript in response to tunicamycin. EBV-immortalized B lymphocytes derived from

AMD patients or case controls, selected from a panel as previously described (Jacobo et al, 2013), were serum-starved for 24h and then cultured in serum-free media that contained tunicamycin (1.2 μM) for 18h. Total RNA was isolated and subjected to gene expression analyses. In one-way ANOVA with Tukey's post-hoc analyses, HtrA1 transcript was increased in normal and AMD subjects (**$p<0.01$). HtrA1 upregulation was not statistically significant between normal and AMD ($p = 0.0559$).

F. Patient-derived lymphocytes from normal or AMD subjects (described in E) displayed differential gene expression for ER stress markers in response to tunicamycin. Total RNA was collected as described above. In response to tunicamycin, lymphocytes derived from normal, but not AMD patients, showed increased expression of total *XBP-1* and *TRIB3*. AMD patient-derived lymphocytes showed increased gene expression for *DDIT3*.

Supplementary Tables

Supplementary Table S1. *HTRA1* silent substitutions rs1049331 (A) and rs2293870 (B) are enriched in all AMD subtypes compared to normal controls. Minor allele frequencies for individuals who harbor a copy of the risk are tabulated below.

A. *HTRA1*

		Normal		Dry AMD		Geographic Atrophy		Neovascular AMD	
		Frequency %	No.	Frequency %	No.	Frequency %	No.	Frequency %	No.
rs1049331	**Genotype**								
	**CC**	53.61%	104	43.27%	45	25.00%	3	28.96%	97
	**TC**	32.47%	63	27.88%	29	8.33%	1	35.82%	120
	**TT**	13.92%	27	28.85%	30	66.67%	8	35.22%	118
	**Total**		194		104		12		335
	**Allele**								
	**C**	69.85%	271	57.21%	119	29.17%	7	46.87%	314
	**T**	30.15%	117	42.79%	89	70.83%	17	53.13%	356
	**Total**		388		208		24		670

B. *HTRA1*

		Normal		Dry AMD		Geographic Atrophy		Neovascular AMD	
		Frequency %	No.	Frequency %	No.	Frequency %	No.	Frequency %	No.
rs2293870	**Genotype**								
	**GG**	40.31%	79	33.67%	33	16.67%	2	21.99%	73
	**TG**	29.59%	58	27.55%	27	8.33%	1	32.53%	108
	**CG**	10.20%	20	9.18%	9	0.00%	0	5.42%	18
	**CT**	4.59%	9	2.04%	2	8.33%	1	4.82%	16
	**TT**	13.78%	27	26.53%	26	66.67%	8	34.64%	115
	**CC**	1.53%	3	1.02%	1	0.00%	0	0.60%	2
	**Total**		196		98		12		332
	**Allele**								
	**G**	60.20%	236	52.04%	102	20.83%	5	40.96%	272
	**T**	30.87%	121	41.33%	81	75.00%	18	53.31%	354
	**C**	8.93%	35	6.63%	13	4.17%	1	5.72%	38
	**Total**		392		196		24		664

Supplementary Table S2. List of oligonucleotide primers used for quantitative real-time RT-PCR

Target human gene	Forward Primer	Reverse Primer
HTRA1	TCCCAACAGTTTGCGCCATAA	CCGGCACCTCTCGTTTAGAAA
GAPDH	CTGGGCTACACTGAGCACC	AAGTGGTCGTTGAGGGCAATG
HPRT	CCTGGCGTCGTGATTAGTGAT	AGACGTTCAGTCCTGTCCATAA
B2M	AAGATTCAGGTTTACTCACGTC	TGATGCTGCTTACATGTCTCG
RPLP0	CATCGTCTTTAAACCCTGCG	TTGTCTGCTCCCACAATGAA
β3-Tubulin	GCGAGATGTACGAAGACGAC	TTTAGACACTGCTGGCTTCG
Unspliced *XBP-1*	CAGCACTCAGACTACGTGC	ATCCATGGGGAGATGTTCTGG
SREBF1	GCCCCTGTAACGACCACTG	CAGCGAGTCTGCCTTGATG
Spliced *XBP-1*	CTGAGTCCGAATCAGGTGCAG	ATCCATGGGGAGATGTTCTGG
Total *XBP-1*	TGGCCGGGTCTGCTGAGTCCG	ATCCATGGGGAGATGTTCTGG
ATF4	GTTCTCCAGCGACAAGGCTA	ATCCTGCTTGCTGTTGTTGG
TRIB3	TACCTGCAAGGTGTACCCC	GGTCCGAGTGAAAAAGGCGTA
DDIT3	AGAACCAGGAAACGGAAACAGA	TCTCCTTCATGCGCTGCTTT

Supplementary Table S3. List of antibodies used in this study

Antibody	Host	Manufacturer	Catalog Number
HRP-conjugated anti-rb IgG	goat	Cell Signaling Technology	7074
HRP-conjugated anti-ms IgG	goat	Cell Signaling Technology	7076
Anti-HtrA1 (for immunoblot) "anti-serine protease/PDZ domains"	Mouse mAb	R & D Systems	MAB2916
Anti-HtrA1 (neutralizing ab) "anti PDZ domain"	Rabbit pAb	Santa Cruz Biotechnology	sc-130780
Anti-HtrA1 (neutralizing ab) "anti-Mac25/KI domains"	Rabbit serum	Homemade (Jacobo, et. al., 2013)	n/a
GRP78/Bip	Rabbit mAb C50B12	Cell Signaling Technology	3177
GRP94β	Rabbit mAb D6X2Q	Cell Signaling Technology	20292
IRE1α	Rabbit mAb 14C10	Cell Signaling Technology	3294
PERK	Rabbit mAb C81H6	Cell Signaling Technology	5683
Phospho-IRE1α	Rabbit pAb	Novus Biologicals	NB100-2323
Ero1-Lα	Rabbit pAb	Cell Signaling Technology	3284
Spliced XBP-1	Rabbit pAb	Santa Cruz Biotechnology	sc-7160
CHOP	Mouse mAb L63F7	Cell Signaling Technology	2895
Phospho-Ser51-eIF2α	Rabbit mAb D9G8	Cell Signaling Technology	3398
CREBH	Mouse mAb	Santa Cruz Biotechnology	sc-377363
SREBP-1	Mouse mAb	Santa Cruz Biotechnology	sc-365513
RasGAP	rabbit	See reference	
Calnexin	Rabbit mAb C5C9	Cell Signaling Technology	2679
Calreticulin	Rabbit mAb	Cell Signaling Technology	12238

	D3E6		
AMFR	Rabbit pAb	Cell Signaling Technology	9590
UHRF1	Rabbit mAb D6G8E	Cell Signaling Technology	12387
USP14	Rabbit mAb D8Q6S	Cell Signaling Technology	11931
PPIA	Rabbit mAb D2Y4M	Cell Signaling Technology	51418
α-tubulin	Rabbit mAb 11H10	Cell Signaling Technology	2125
Acetylated Lysine40-α-tubulin	Rabbit mAb D20G3	Cell Signaling Technology	5335
Alexa488 anti-rabbit (Fab)2	goat	Jackson Immunoresearch	111-546-003
Alexa594 anti-mouse (Fab)2	goat	Jackson Immunoresearch	111-586-003
Vimentin	rabbit	Abcam	

Supplementary Dataset S1. Collection of chordate HtrA protein sequences used in phylogenetic analyses.

```
>Anolis_carolinensis-ENSACAP00000008508.2-HTRA2
S---------------------------------------------------P-------
------------------------------------------------------------
------------------------------------------------------------
------------------------------------------------------------
------------------------------------------------------------
---------------------------------------PPPRWRWAAMGACGAGAGLLL
LLF-------------------------------------FP--------RKPAEA----
-RPP---V--P-S-PPPSPRSTYNFIADVVERTAPALVYIEIL-GR--HPF----SGREV
PISNGSGFVVSSDGLIVTNAHVVAN-------RR---RVRVRLASGELYDATVRLVDQVA
DIATLKI-S---------------------PK-----------------------KPLPT
LRLGRSSEVRQGEFVVAMGSPFALQNTITSGIVSSAQRGGRELGLSS---SDIE-YIQTD
AAID--FGNSGGPLVNLDGE--VIGVNTMKVTPGISFAIPSDRLRAFLEKEEKSKDSWF-
G--G-----------K--------------------KEGKRRYIGVMMLTLTS-------
------------------------------------------------------------
------------------------------------------------------------
------------------------------------------------------------
-------S----------------------------------------------------
------------------------------------------------------------
------------------------------------------------------------
------------------------ILSELKMRDPSFPDISY--GVLIHKVIIGSPAHQAG
LKAGDVVVEINGRASRRAEDVYEAVRTQ-DHLTLLVHRGYEALLLTITPE-----V----
--TE
>Anolis_carolinensis-ENSACAP00000008914.2-HTRA4
------------------------------------------------------------
------------------------------------------------------------
------------------------------------------------------------
------------------------------------------------------------
------------------------------------------------------------
------------------------------------------------------------
------------------------------------------------------------
-CSA---D--T-P-DPNSLRQRFNFIADVVEKIAPAVVHLELF-RR--VPY----TNKEI
LVSSGSGFIVSEDGLIITNAHVLTN-------KQ---RIKVELKSGHQFDAKIKEVDHKL
DIALIKI-D---------------------AN-----------------------MDLPV
LLLGRSSDLRPGEFVVALGSPFSLQNTVTTGIVSSTQRDGKELGLKD---SDME-YIQTD
AIIN--YGNSGGPLVNLDGE--VIGMNTLKVTAGISFAIPSDRIRQFLEDSHN---RQT-
K--G-----------K--------------------SRTKKKYLGLRMLPLTF-------
------------------------------------------------------------
------------------------------------------------------------
```

-------N--

------------------------LIRELRRRDRNFPDLIAGVGVYVYEVIPGTAAESAG
LEDGDVIIAINGKTVTSTRDVTDAVQNS-EALAIVVRRGNEDLILTVVPD----E-----
--ID
>Anolis_carolinensis-ENSACAP00000011769.2-HTRA3

--------------------------PA-GDRCPARCDVSRCP-S-S--ICPSG------
-Y--VPD-GCNCCLVCAAGEGEACGR----A---E----D-PPCGEGLQCRSFNA-----
------------E--P--ERRG-VCQCKL-G---AAVCGSDGRS-YANAC---------R
LQT-------------------------------------AARQALQQ-GRPPVRQVQKG
ACRAASTG--H-Q-QLNSPRFKFNFIADVVEKIAPAVVHIELF-LR--HPL----FGHNV
PLSSGSGFIMSDTGLIVTNAHVVSSSS-TVTGRQ---QLKVQLQDGDTYEAKIKDIDKKS
DIATIKI-N---------------------PR-----------------------KKLPA
LSIGQSGDLRPGEFVVAMGSPFALQNTVTTGIVSTAQRDGKELGLKD---SDMD-YIQTD
AIIN--YGNSGGPLVNLDGE--VIGINTLKVTAGISFAIPSDRIAQFLSENYD---KKG-
N--S-----------K--------------------IDGKKRFIGIRMLTITP-------

-------V--

------------------------LMEELKVNNPDFPDVIS--GIYVHEVVPNSPSQRGG
IEDGDIIVKVNGRPLKTSADLQDAVMNE-SPLLLEVRRGNDDLLFNIEPE----VV----
--IP
>Anolis_carolinensis-ENSACAP00000017127.3-HTRA1
M---K-------
---R------------
------L----------------S--------------------------F--------L
LLL-L--AALTEAQLH----GR-RFEDR-AAPCPDRCDRSRCP-ELP-ADCAGS------
-P--ALD-PCGCCPVCAAQEGEPCWA----T---AG--GELPSCAEGLLCVLSPGEGGAP
------ASATVRR--R--AKAG-RCACAS-P---EPVCGSDGET-YTSLC---------Q
LRT-------------------------------------ASRRSEGH-GQPALIAIQRG
PCSH---G--Q-E-DPNSLRHKYNFIADVVEKIAPAVVHIELF-RK--LPF----SKREV
IVASGSGFIVSEDGLIVTNAHVVTN-------KN---KVKVELKNGVTYEAKIKDVDEKA
DIALIKI-D---------------------TQ-----------------------GKLPV
LLLGRSAELRPGEFVVAIGSPFSLQNTVTTGIVSTTQRGGKELGLRN---SDMD-YIQTD
AIIN--YGNSGGPLVNLDGE--VIGINTLKVTAGISFAIPSDKIKKFLTESHD---RQS-

```
K--G-----------K--------------------AVTKKKYIGIRMVSLTP-------
------------------------------------------------------------
------------------------------------------------------------
------------------------------------------------------------
-------S----------------------------------------------------
------------------------------------------------------------
------------------------------------------------------------
------------------------KAKELKERHKDFPDVIS--GAYIIEVIPGTPAEAGG
LKENDIIISINGKPILSANDVSDIIKKD-NTLSLVVRRGNEDIILTVIPE----EI----
--DD
>Canis_familiaris-ENSCAFP00000008772.3-HTRA4
------------------------------------------------------------
------------------------------------------------------------
------------------------------------------------------------
------------------------------------------------------------
------------------------------------------------------------
----------PLR--G--ARGG-TCGCPA-G---AAVCGSDGRT-YPSLC---------E
LRA-------------------------------------QNRAARLR-GALPAVPVLKG
DCAG--RE--A-W-SVGRLRSQYNFLAAVVEKVAPSVVHLQLF-RR--SPL----SSKDM
PASSGSGFIVSEDGLIVTNAHVITN-------QQ---RIQVELQSGVQYEATIKDIDHKL
DLALIKI-E---------------------PN-----------------------GDLPV
LLLGRSSDLQAGEFVVALGSPFSLQNTVTAGIVSTTQRGGRELGLKD---SDMD-YIQTD
AIIN--HGNSGGPLVNLDGD--VIGINTLKVTAGISFAIPSDRIRQFLAEFHE---RQL-
K--G-----------K--------------------ALSQKKYLGLRMLPLTM-------
------------------------------------------------------------
------------------------------------------------------------
------------------------------------------------------------
-------N----------------------------------------------------
------------------------------------------------------------
------------------------------------------------------------
------------------------LLQEMKRQDPDFPDVSS--GVFVYEVIQGTAAESSG
LRDHDVIVSINGQPVTTTTDVIEAVKDS-DSLSIMVLRGSQTLILTVTPE----I-----
--IN
>Canis_familiaris-ENSCAFP00000012325.3-HTRA2
M---------------------------------------------------A-------
-----------------------------------------------A------------
------L----------------RAGRGAAWNLRGWRALGGVHSGKGPLLT--------P
D-L-RALLTS--GIPD----PR-ARVTYGTPSLRARWSVGVP---EP-RTCLTS------
-R--TSDSRAWLAVG---------------------------------------------
--------------------TP-DPRTQE-G---SGTPGTRPRIWLAVALGAGGAVLLLL
WGG-------------------------------------GR--------GPPAVL----
-ASV---L--G-S-PPSSPRSQYNFIADVVEKTAPAVVYIEIL-GR--HPF----SGREV
PISNGSGFVVAADGLIVTNAHVVAD-------RR---RVRVRLLSGDTYEAVVTAVDPVA
```

```
DIATLRI-Q---------------------TK-----------------------EPLPT
LPLGRSADVRQGEFVVAMGSPFALQNTITSGIVSSAQRPARDLGLPQ---TNVE-YIQTD
AAID--FGNSGGPLVNLDGE--VIGVNTMKVTAGISFAIPSDRLREFLHRGEK---KNSW
F--G-----------I--------------------SGSQRRYIGVMMLTLTP-------
------------------------------------------------------------
------------------------------------------------------------
------------------------------------------------------------
-------S----------------------------------------------------
------------------------------------------------------------
------------------------------------------------------------
------------------------ILAELQLREPSFPDVQH--GVLIHKVILDSPAHRAG
LRPGDVILAIGEQLVQNAEDIYEAVRTQ-SQLAVRIRRGPETLTLYVTPE-----V----
--TE
>Canis_familiaris-ENSCAFP00000021445.3-HTRA3
------------------------------------------------------------
------------------------------------------------------------
------------------------------------------------------------
------------------------------------------------------------
-------------------------R----L---L----V-SPCEKSVECA---------
--------------------RG-VFRCGW-A---HPVCGTDGHT-YANVC---------A
LQA-------------------------------------ASRRALEL-SGTPVRQLQKG
ACPS---G--L-H-QLTSPRYKFNFIADVVEKIAPAVVHIELF-LR--HPL----FGRNV
PLSSGSGFIMSEAGLIVTNAHVVSSTN-AVSGRQ---QLKVQLQNGDTYEATIKDIDKKS
DIATIKI-H---------------------PK-----------------------KKLPA
LLLGRSADLRPGEFVVAIGSPFALQNTVTTGIVSTAQRDGKELGLRD---SDMD-YIQTD
AIIN--YGNSGGPLVNLDGE--VIGINTLKVAAGISFAIPSDRITRFLTEFQD---KHV-
K------------------------------------DWKKRFIGIRMRTITP-------
------------------------------------------------------------
------------------------------------------------------------
------------------------------------------------------------
-------S----------------------------------------------------
------------------------------------------------------------
------------------------------------------------------------
------------------------LVEELKASNPDFPSVSS--GIYVQEVVPNSPSQRGG
IQDGDIIVKVNGRPLADSSELQEAVLTE-SPLLLEVRRGNDDILFSISPE-----V----
--VL
>Canis_familiaris-ENSCAFP00000039791.1-HTRA1
M---------------------------------------------------G-------
-----------------------------------------------V------------
------A-----------------------------------------------------
---------------------------------------------SY-LSVWSL------
-E--SLEDLCNRVQV---------------------------------------------
--------------------KT-PDLVLS-S---LVQGSKGPGVYFGSFSPMPFSGGHST
```

```
EHS-------------------------------------VL--------TCPVFA----
-LFS---G--Q-E-DPNSLRHKYNFIADVVEKIAPAVVHIELF-RK--LPF----SKREV
PVASGSGFIVSEDGLIVTNAHVVTN-------KH---RVKVELKNGATYEAKIKDVDEKA
DIALIKI-D---------------------HE-----------------------GRLPV
LLLGRSSELRPGEFVVAIGSPFSLQNTVTTGIVSTTQRGGKELGLRN---SDMD-YIQTD
AIIN--YGNSGGPLVNLDGE--VIGINTLKVTAGISFAIPSDKIKKFLTESHD---RQA-
K--G-----------K--------------------AITKKKYIGIRMMSLTS-------
------------------------------------------------------------
------------------------------------------------------------
------------------------------------------------------------
-------S----------------------------------------------------
------------------------------------------------------------
------------------------------------------------------------
------------------------KAKELKDHHRDFPDVLS--GAYIIEVIPDTPAEAGG
LKENDVIISINGQSVVSASDVSDVIKKE-NTLNMVVRRGNEDIMITVIPE----EI----
--DP
>Ciona_intestinalis-ENSCINP00000035284.1-
------------------------------------------------------------
------------------------------------------------------------
------------------------------------------------------------
------------------------------------------------------------
------------------------------------------------------------
------------------------------------------------------------
-----------------------------------------------------------G
---A---G--N-T-GGNTRRGAFNFVADVVQVAGPAVVHIERL-VN--VPF----TRRAV
AVSNGSGFLVESNGLIITNAHVVGN-------QS---ALNVKLQDGREFEGQVIGIDEAR
DIAAVKI-N---------------------CT------------------------GLPK
IPLGTTRDLRPGEWVVAIGSPLALKNTVTAGIVSNMCRAGKELGLRDEEKRDME-YIQTD
ATIN--VGNSGGPLVNLDGE--VIGINSMMASAGIGFAIPIDYVRDFLQQLEH---GQQ-
---T-----------R--------------------VQHTRRWIGVSMLSLTP-------
------------------------------------------------------------
------------------------------------------------------------
------------------------------------------------------------
-------E----------------------------------------------------
------------------------------------------------------------
------------------------------------------------------------
------------------------ISSHLRARNPDFPDVTT--GVYVHKVTPDSPSDIAG
VQNGDVIIKINGKQINGVSDVLETLKQN-QVLNASICRRGRTIVVRIIAE----EI----
--VH
>Ciona_savignyi-ENSCSAVP00000012890.1-
M---------------------------------------------------A-------
-----------------------------------------------A------------
------F----------------T------------------------------------
```

```
---------------------------------------RKC---------ANGVIKSGL
IT--NLL-EHRVCAPPLTAFYS-----------------------------SYKS-----
---------NSEN--S--NGENKNSKWKV-A---ICVTGA------LGAY---------I
THK-------------------------------------SRSNHVSAARYDPSSP-FRG
---A---G--N-T-GSNTRRGAFNFIADVVQVAGPAVVHIERL-VN--VPF----TRKAI
AVSNGSGFIVDSNGLIVTNAHVVGN-------QS---ALKVKLQDGREFEGQVIGIDEAR
DIAAVKV-N---------------------CT------------------------QLPQ
IPLGTTRDLRPGEWVVAIGSPLALQNTVTAGIVSNMCRGGKELGLRDEEKRDME-YIQTD
ATIN--VGNSGGPLVNLDGQ--VIGVNSMMASAGIGFAIPIDYVREFLQQLEH---GQQ-
---T-----------R--------------------VRHSRRWIGINMLSITP-------
------------------------------------------------------------
------------------------------------------------------------
------------------------------------------------------------
-------D----------------------------------------------------
------------------------------------------------------------
------------------------------------------------------------
------------------------IASHLRSRNPDFPDVTS--GVYVHKVQYESPSYMAG
VQDGDVITDINGQPIQGVGDVLEALKHS-QVLNAKIRRKSRTIVIRIVAE----EI----
--VH
>Danio_rerio-ENSDARP00000016871.8-HTRA3
------------------------------------------------------------
------------------------------------------------------------
------------------------------------------------------------
------------------------------------------------------------
------------------------------------------------------------
------------------------------------------------------------
------------------------------------------------------------
------------------------------------------------------------
---------MSETGLIVTNAHVVSSTT-SVSGHQ---RLKVQMRDGDVYEATIQDIDKKS
DIATIKI-N---------------------PQ-----------------------KKLPV
LLLGHSADLRPGEFVVAIGSPFALQNTVTTGIVSTAQRDGKELGLQD---SDMD-YIQTD
AIIN--YGNSGGPLVNLDGE--VIGINTLKVAAGISFAIPSDRITRFLNDSLG---KQN-
K--E-----------T--------------------RSVKKRFIGIRMLTITD-------
------------------------------------------------------------
------------------------------------------------------------
------------------------------------------------------------
-------A----------------------------------------------------
------------------------------------------------------------
------------------------------------------------------------
------------------------LVEELKQQNPDFPDVSS--GIFVHEVVPHSPAQKGG
IRDGDIIVKLNGEPLLSTSDLKEALNQD-MTLLLEVRRGNDDLLFNIEPD----II----
--MQ
>Danio_rerio-ENSDARP00000020528.6-htra1b
```

```
M---------------------------------------------------R-------
----------------------------------------------L------------
------L----------------I--------------------------L---------
C-A-SIILVP--LLCD----AR-IIKRY-VIGCPERCDKSLCP-PIP-PDCLAG------
-D--ILD-QCDCCPVCAAGEGESCGG----TGKLG----D-PECGEGLECAVSDG---VG
------ATTTVRR--R--GKTG-VCVCKS-S---EPVCGSDGVS-YRNIC---------E
LKR-------------------------------------VSNRAQKL-QQPPIIFIQRG
ACGK---GH-E-E-NPDSLRHRYNFIADVVEKIAPAVVHIELF-RK--NVF-----NREV
AVASGSGFVVSEDGLIVTNAHVVAN-------KH---RVKVELKTGTTYDAKIKDVDEKA
DIALIKI-D----------------------AP-----------------------MKLPV
LLLGRSADLRPGEFVVAIGSPFSLQNTVTTGIVSTTQRGGKELGLRN---SDMD-YIQTD
AIIN--YGNSGGPLVNLDGE--VIGINTLKVTAGISFAIPSDKIRQFLAESHD---RQA-
K--G-----------K--------------------TATKKKYIGVRMMTLTP-------
------------------------------------------------------------
------------------------------------------------------------
------------------------------------------------------------
-------T----------------------------------------------------
------------------------------------------------------------
------------------------------------------------------------
------------------------LAKELKQRKNDFPDVTS--GAYVIEVIPKTPAEVGG
LKESDVIISINGQRITSASDVSTAIKTD-ESLRAVVRRGNEDIILTIIPE----EI----
--DP
>Danio_rerio-ENSDARP00000048135.5-htra1a
M---------------------------------------------------I-------
----------------------------------------------L------------
------V----------------T--------------------------L--------F
C-I-CALVTS--LQAD----AR-LSKRYVIGGCPSHCDKSMCP-TMP-KDCSTG------
-Q--VMD-HCNCCLVCASGEGEACGG----VGKLG----D-PVCGESLECSVTGG---VS
------YSATVRR--R--GKQG-VCVCKS-S---DPVCGSDGVS-YRDIC---------E
LKR-------------------------------------VSNRAQSL-QQPPVLFIQRG
ACGT---SL-H-D-NPNSLRYKYNFIADVVEKIAPAVVHIELY-RK--MVY----SKREM
AVASGSGFVVSDDGLIVTNAHVVAN-------KN---RVKVELKNGASYDAKIKDVDEKA
DIALIKI-D----------------------LP-----------------------NKLPV
LLLGRSADLRPGEFVVAIGSPFSLQNTVTTGIVSTTQRGGKELGLRN---SDMD-YIQTD
AIIN--YGNSGGPLVNLDGE--VIGINTLKVTAGISFAIPSDKIRQFLAESYD---RLA-
R--G-----------R--------------------GTTKKRYIGVRMMTLTP-------
------------------------------------------------------------
------------------------------------------------------------
------------------------------------------------------------
-------S----------------------------------------------------
------------------------------------------------------------
------------------------------------------------------------
------------------------LSKELKGRLRDFPDITS--GAYVIEVISKTPAAAGG
```

LKEHDVIISINGQRISTATDVSAIIKKE-SSLRVVVRRGNEDIILTIIPM----EI----
--DP
>Danio_rerio-ENSDARP00000050541.6-htra4
M---M-------
---W------------
------K----------------L--------------------------L--------A
CVF-M--A----STLH----AR-VLRKR-HSTCAHQCDLSRCS-SSL-EYCYFG------
-V--VMD-SCGCCSVCAAGEGDYCGE----D---R----L-GVCGAGMTCEERS------
------------------AERS-TCVCDS-H---EPVCGSDGRT-YPSIC---------R
LKA-------------------------------------ENRRAKTS-GIPAVIFIQRG
PCET---G--S-R-NPSSMRYKFNFIADVVDKIAPAVVHLELF-SR--LPF----SNQDV
PVSSGSGFIVSEDGWIVTNAHVLSN-------KQ---RIKVELKNGMLYDATIKDVDQKL
DIALIKI-D---------------------SE-----------------------TALPV
LLLGRSSDLRPGEFVVAVGSPFSLQNTVTTGIISTTHRGGHELGLQN---SDME-YIQTD
AIIN--YGNSGGPLVNLDGD--VIGINTLKVTPGISFAIPSDRIRQFLADSYE---QRR-
K--G-----------R--------------------TLTKKKYMGVRMLQLSA-------

-------A--

------------------------LIRELREKESSFPDVSS--GVYVYEVIPGTAAFSAG
MLNQDVIISINGQPVYSTEDVSQAVQSN-EILSLMVRRAQKEITLTVVPG----E-----
--VD
>Danio_rerio-ENSDARP00000075010.4-htra3a
M---Q-------
---V------------
------I----------------L--------------------------L--AAA---T
FIF------------T----QDLAIAEL-KSNCPSKCDVSKCP-S-P--SCPSG------
-Y--VPD-RCNCCLVCSLGEGAPCGR----K---E----D-VLCGDGLECKFPTG-----
------------K--R--LSKG-VCQCKT-I---HRVCGTDGKT-YGNVC---------K
MRT-------------------------------------ASRKAQQR-RLSVITQRHKG
SCAPSSSD--P-A-HLNSPRYKFNFIADVVEKIAPAVVHVELF-LN--HPL----FGRHV
PLSSGSGFIMTQSGLIVTNAHVVASSA-TVTGRQ---HLRVQLHDGQTYEASIRDIDKKS
DIATIKI-N---------------------PK-----------------------KKLQV
LSLGRSADLRPGEFVVAIGSPFALQNTVTTGIVSTTQRDGKELGIRD---SDMG-YIQTD
AIIN--YGNSGGPLVNLDGE--VIGINTLKVTAGISFAIPSDRINKFLDESND---KQQ-
K--V-----------KQRVVRTNYTQSQAMRTASDVNVPMKRFIGIKMVTLTE-------

-------N--

------------------------LVHELKWHNPAFPDIGS--GILVHEVIADSPAQKGG
LESGDIIVKLNGHPLMNTGELQEAIQVD-MPLLLEVRRGNDDLLFNIEPQ----IL----
--MQ
>Danio_rerio-ENSDARP00000092827.4-HTRA2

--MCVKV---
------------NLRLPWGAPFLLK---TPSHLASSQRGSKELGLSN---SNMD-YIQTD
ATID--FGNSGGPLINLDGE--VIGINTMKVTAGISFAIPL-----FLDRSAD---KQK-
SWFG-----------E--------------------SESKRRYIGVMMLTLTP-------

-------S--

------------------------IIKELRMRDPSFPDVSH--GVLIHRVIVGSPANRAG
MKPGDVIIEINGVKVNTSEEIYNAVRTS-ESLNVVVRRGADLLMLHMTPE-----S----
--TE
>Dasypus_novemcinctus-ENSDNOP00000012617.3-HTRA1
MGWAARPRALAPAPRRAQTRPPRRHRLPRSPRRRCPALAAPAAAVGAAMQSPR-------
---A------------
------A----------------L----------------------------LA-LVPPL
LLL-L--AAP--ASAQA-SRAG--RSVP-ATGCPERCDPARCP-PSS-EHCEGD------
-R--VRD-ACGCCEVCGAPEGAACGL----Q--------E-GPCGEGLQCVVPFG---VP
------ASATVRR--R--AQAG-LCVCTS-S---EPVCGSDANT-YANPC---------Q
LRA-------------------------------------ASRRSERL-SQPPVIVLQRG
ACGQ---G--Q-E-DPNSLRHKYNFIADVVEKIAPAVVHIELF-RK--LPF----SKREV
PVASGSGFIVSEDGLIVTNAHVVTN-------KH---RVKVELKNGATYEAKIKDVDEKA
DIALIKI-D---------------------HQ-----------------------GKLPV
LLLGRSSELRPGEFVVAIGSPFSLQNTVTTGIVSTTQRGGKELGLRN---SDMD-YIQTD
AIIN--YGNSGGPLVNLLFRRGSHWINTLKVTAGISFAIPSDKIKKFLTESHD---RQA-
K--G-----------K--------------------AITKKKYIGIRMMSLTS-------

```
------------------------------------------------------------
------------------------------------------------------------
-------S----------------------------------------------------
------------------------------------------------------------
------------------------------------------------------------
------------------------KAQELRDRHRDFPDVLS--GAYIIEVIPDTPAEAGG
LKENDVIISINGQSVVSANDVSDVIKKE-STLNMVVRRGNEDIMVTVIPE----EI----
--DP
>Dasypus_novemcinctus-ENSDNOP00000015442.3-HTRA2
M---------------------------------------------------A-------
-----------------------------------------------A------------
------L----------------RAGRAAGRSLWGWRALGGARWGTGPVVT--------P
D-L-RALLTS--GASD----AR-ARVTYWPLRPPARLSAGDP---EP-PACPTS------
-G--TPD-PPRARLT---------------------------------------------
--------------------AG-TSASRN-A---SETPGARPPVWLAVALGAGGAVVLLL
WGG-------------------------------------GR--------GPPAVL----
-AAV---P--G-P-PPTSPRSQYNFIADVVEKTAPAVVYIEIL-GR--HPF----SGREV
PISNGSGFVVAADGLIVTNAHVVAD-------RR---RVRVRLPSGDTYEATVTAVDPVA
DIATLRI-Q---------------------TK-----------------------EPLPT
LPLGRSSDVRQGEFVVAMGSPFALQNTITSGIVSSAQRPARDLGLPQ---TNVE-YIQTD
AAID--FGNSGGPLVNLDGE--VIGVNTMKVTAGISFAIPSDRLREFLHRGEK---KNSW
F--G-----------I--------------------SGSQRRYIGVMMLTLTP-------
------------------------------------------------------------
------------------------------------------------------------
------------------------------------------------------------
-------S----------------------------------------------------
------------------------------------------------------------
------------------------------------------------------------
------------------------ILAELQLREPSFPDVQH--GVLIHKVILGSPAHRAG
LRPGDVILAIGEQLVQNAEDVYEAVRTQ-SQLAVRIRRGPETLTLYVTPE-----V----
--TE
>Dasypus_novemcinctus-ENSDNOP00000023192.1-HTRA4
I---------------------------------------------------D-------
-----------------------------------------------V------------
----GLG----------------T--------------------------A--------L
H-C-LGVGLV--PGGG----VG-IWGCV-CRGLPDSCEPTRCP-PLP--SCPAG------
-APPVPD-RCGCCRVCPAAEGEACGG----A---R----A-RPCAPGLQCGAPPG---P-
-----------RRFGG--AAPG-TCGCAA-A--GAAVCGSDGRT-YRSLC---------A
LRA-------------------------------------ENRAARLR-GALPAVPVQKG
GCGE--RG--T-R-SAGRLRSAHNFIAAVVEQVAPSVVHLQLF-RRDRSPL----SGKDL
PASSGSGFLVSEDGLIVTNAHVITN-------QQ---RIQVELQSGIQYEATVKDVDHKL
DLALIKI-E---------------------PD-----------------------TDLPV
LLLGRSSDLRAGEFVVALGSPFSLX--VIPGIRDIGGLGGTGVHWRR---LLLV-NLFTE
```

LCINDEHGNSGGPLVNLDGD--VIGINTLKVTAGISFAIPSDRIRQFLAEFHE---RQL-
K--G-----------K--------------------ALSQKKYLGLRMLPLSM-------

-------N--

------------------------LLQEMKRQDPDFPDVSS--GVFVYEVIQGTAAESSG
LRDHDVIVSINGQPVSTTTDVAEAVKDN-DSLSIMVRRGSQTLILTVTPE----II----
--KP
>Dasypus_novemcinctus-ENSDNOP00000033323.1-HTRA3
R---GKAARPVA
LCPGACGKRAPPPPRHLNPRLLCAQVACVFTRVRAVVPRATLQAWAEA------------
------R----------------A--------------------------L--L------
----L--AAL----AA----LALAREPP-APSCPARCDVSRCP-S-P--RCPGG------
-Y--VPD-PCNCCLLCAASEGEPCGR----P---L----D-SPCGDSLECV---------
--------------------RG-ACRCRG-A---HAVCGTDGHT-YANVC---------A
LQA-------------------------------------ASRRALQL-FGTPVRQLQKG
ACPS---G--L-H-QLTSPRMKFNFIADVVEKIAPSVVHIELF-LR--HPL----FGRNV
PLSSGSGFIVSEAGLIITNAHVVSNSN-GVSGRQ---QLKVQLQNGDSYEAAIQDIDKKS
DIATIKI-H---------------------PQ-----------------------RKLPV
LLLGRSADLRPGEFVVAIGSPFALQNTVTTGIVSTAQRDGRELGLRD---SDMD-YVQTD
AIIN--YGNSGGPLVNLDNN--CLSINTVHLSHRFSSLIPLWNMVRLLGNNSS---LVA-
R--A-----------RA------------------LLEWKKRFIGIRMRTVTP-------

-------S--

------------------------LVEELKAGNPDFPAVSS--GIYVQEVVPKSPAERGG
IQDGDLIVKVNGRALADSSELQEAILTE-SPLLLEVRRGNDDLLFSIAPE-----V----
--VV
>Gallus_gallus-ENSGALP00000025068.4-HTRA3
M---R-------
---L------------
------S----------------L--------------------------L--SPL----
LLLCL--SRS----RG----SAELP-AA-RAKCPTRCDVSKCP-S-P--SCPSG------
-Y--VPD-RCNCCLICAAGEGDSCGR----K---E----D-PPCGDSLDCRYPMG-----
------------K--R--FAKG-VCQCKL-S---VQVCGSDGRT-YDNIC---------Q
LKA-------------------------------------VSRKALQH-GLPAVAQVQKG
ACES---G--H-L-QSSSPRYKFNFIADVVEKIAPAVVHIELF-LR--HPL----FGRNV

PLSSGSGFIMSDSGLIVTNAHVVSSTN-AISGRQ---QLKVQLQNGDTYEATIRDIDKKS
DIATIKI-H---------------------PK-----------------------KKLPV
LLLGHSADLRPGEFVVAIGSPFALQNTVTTGIVSTAQRDGKELGLRD---SDMD-YIQTD
AIIN--YGNSGGPLVNLDGE--VIGINTLKVTAGISFAIPSDRITQFLTESLD---KQN-
K------------------------------------DSKKRFIGIRMLTITP-------

-------A--

------------------------LVEELKHNNADFPDVRS--GIYVHEVVPNSPSHRGG
IQDGDIIVKVNGRPLMTSSDLQEAVMNE-SPLLLEVRRGNDDLLFNIEPE-----I----
--VM
>Gallus_gallus-ENSGALP00000025070.4-HTRA1

---XRRAERL-RQPPIIAIQRG
GCGQ---G--Q-E-HPNSLRHKYNFIADVVEKIAPAVVHIELF-RK--LPY----SKREI
PVASGSGFIVSEDGLIVTNAHVVTN-------KN---RVKVELKNGETYEAKIKDVDEKA
DIALIKI-D---------------------AQ-----------------------GKLPV
LLLGQSGDLRPGEFVVAIGSPFSLQNTVTTGIVSTTQRGGKELGLRN---SDMD-YIQTD
AIIN--YGNSGGPLVNLDGE--VIGINTLKVTAGISFAIPSDKIKKFLTESHD---RQA-
K--G-----------K--------------------AITKKKYIGIRMMSLTP-------

-------S--

------------------------KAGELKDRHKDFPDVVS--GAYVIDVIPETPAEAGG
LKDNDVIISINGQSITSASDVSDIIKKD-STLNMVVRRGNEDVMLTVIPE----EI----
--DP
>Gallus_gallus-ENSGALP00000042257.1-HTRA2
M---Q-------
---L------------
------P---

------------------AALASLIPAGPLRVSTPS-CDLCPP-CR--HPF----SGREV
PISNGSGFLVSPDGLIVTNAHVVAN-------RR---RVRVKLASGEQYDAVVQDVDQVA
DIATIRI-K---------------------PKVRAAAREGSLPRLPSAYTVPLFQHPLPT
LPLGRSSEVRQGEFVVAMGSPFALQNTITSGIVSSAQRGSRELGLAA---SDME-YIQTD
AAID--FGNSGGPLVNLDGE--VIGVNTMKVTSGISFAIPSDRLRKFLQKEEE---RKS-
SWFG-----------N--------------------AETKRRYIGVMMLTLTP-------

-------S--

------------------------ILAELKLRDPSFPDVSY--GVLIHKVIIGSPAHQAG
LKAGDVVLEINGQATRRAEDVYEAVRTQ-QSLALLVRRSYDTLL---EPG-----T----
--QD
>Homo_sapiens-ENSP00000258080.3-HTRA2
M---A-------
---A------------
------P----------------RAGRGAGWSLRAWRALGGIRWGRRPRLT--------P
D-L-RALLTS--GTSD----PR-ARVTYGTPSLWARLSVGVT---EP-RACLTS------
-G--TPGPRAQLTAV---
--------------------TP-DTRTRE-A---SENSGTRSRAWLAVALGAGGAVLLLL
WGG-------------------------------------GR--------GPPAVL----
-AAV---P--S-P-PPASPRSQYNFIADVVEKTAPAVVYIEIL-DR--HPF----LGREV
PISNGSGFVVAADGLIVTNAHVVAD-------RR---RVRVRLLSGDTYEAVVTAVDPVA
DIATLRI-Q---------------------TK----------------------EPLPT
LPLGRSADVRQGEFVVAMGSPFALQNTITSGIVSSAQRPARDLGLPQ---TNVE-YIQTD
AAID--FGNSGGPLVNLDGE--VIGVNTMKVTAGISFAIPSDRLREFLHRGEK---KNSS
S--G-----------I--------------------SGSQRRYIGVMMLTLSP-------

-------S--

------------------------ILAELQLREPSFPDVQH--GVLIHKVILGSPAHRAG
LRPGDVILAIGEQMVQNAEDVYEAVRTQ-SQLAVQIRRGRETLTLYVTPE-----V----
--TE
>Homo_sapiens-ENSP00000303766.2-HTRA3
M---Q-------
---A------------

```
------R----------------A--------------------------L--L------
----L--AAL----AA----LALAREPP-AAPCPARCDVSRCP-S-P--RCPGG------
-Y--VPD-LCNCCLVCAASEGEPCGG----P---L----D-SPCGESLECV---------
--------------------RG-LCRCRW-S---HAVCGTDGHT-YANVC---------A
LQA-------------------------------------ASRRALQL-SGTPVRQLQKG
ACPL---G--L-H-QLSSPRYKFNFIADVVEKIAPAVVHIELF-LR--HPL----FGRNV
PLSSGSGFIMSEAGLIITNAHVVSSNS-AAPGRQ---QLKVQLQNGDSYEATIKDIDKKS
DIATIKI-H---------------------PK-----------------------KKLPV
LLLGHSADLRPGEFVVAIGSPFALQNTVTTGIVSTAQREGRELGLRD---SDMD-YIQTD
AIIN--YGNSGGPLVNLDGE--VIGINTLKVTAGISFAIPSDRITRFLTEFQD---KQI-
K------------------------------------DWKKRFIGIRMRTITP-------
------------------------------------------------------------
------------------------------------------------------------
------------------------------------------------------------
-------S----------------------------------------------------
------------------------------------------------------------
------------------------------------------------------------
------------------------LVDELKASNPDFPEVSS--GIYVQEVAPNSPSQRGG
IQDGDIIVKVNGRPLVDSSELQEAVLTE-SPLLLEVRRGNDDLLFSIAPE-----V----
--VM
>Homo_sapiens-ENSP00000305919.4-HTRA4
M---------------------------------------------------I-------
-----------------------------------------------R------------
------P---------------QL---------------------RTAGLGRC-LLPGL
LLL-L--V--PVLWAG----AE-K-LHT-QPSCPAVCQPTRCP-ALP--TCALG------
-TTPVFD-LCRCCRVCPAAEREVCGG----A---Q----G-QPCAPGLQCLQPL------
------------R--P--GFPS-TCGCPTLG---GAVCGSDRRT-YPSMC---------A
LRA-------------------------------------ENRAARRL-GKVPAVPVQWG
NCGD--TG--T-R-SAGPLRRNYNFIAAVVEKVAPSVVHVQLW-GR--LLH----GSRLV
PVYSGSGFIVSEDGLIITNAHVVRN-------QQ---WIEVVLQNGARYEAVVKDIDLKL
DLAVIKI-E---------------------SN-----------------------AELPV
LMLGRSSDLRAGEFVVALGSPFSLQNTATAGIVSTKQRGGKELGMKD---SDMD-YVQID
ATIN--YGNSGGPLVNLDGD--VIGVNSLRVTDGISFAIPSDRVRQFLAEYHE---HQM-
K--G-----------K--------------------AFSNKKYLGLQMLSLTV-------
------------------------------------------------------------
------------------------------------------------------------
------------------------------------------------------------
-------P----------------------------------------------------
------------------------------------------------------------
------------------------------------------------------------
------------------------LSEELKMHYPDFPDVSS--GVYVCKVVEGTAAQSSG
LRDHDVIVNINGKPITTTTDVVKALDS--DSLSMAVLRGKDNLLLTVIPE----T-----
--IN
```

```
>Homo_sapiens-ENSP00000357980.3-HTRA1
M---------------------------------------------------Q-------
-----------------------------------------------I------------
------P----------------R----------------------------AA-LLPLL
LLL-L--AAP--ASAQLSRAGR--SAPL-AAGCPDRCEPARCP-PQP-EHCEGG------
-R--ARD-ACGCCEVCGAPEGAACGL----Q--------E-GPCGEGLQCVVPFG---VP
------ASATVRR--R--AQAG-LCVCAS-S---EPVCGSDANT-YANLC---------Q
LRA-------------------------------------ASRRSERL-HRPPVIVLQRG
ACGQ---G--Q-E-DPNSLRHKYNFIADVVEKIAPAVVHIELF-RK--LPF----SKREV
PVASGSGFIVSEDGLIVTNAHVVTN-------KH---RVKVELKNGATYEAKIKDVDEKA
DIALIKI-D---------------------HQ-----------------------GKLPV
LLLGRSSELRPGEFVVAIGSPFSLQNTVTTGIVSTTQRGGKELGLRN---SDMD-YIQTD
AIIN--YGNSGGPLVNLDGE--VIGINTLKVTAGISFAIPSDKIKKFLTESHD---RQA-
K--G-----------K--------------------AITKKKYIGIRMMSLTS-------
------------------------------------------------------------
------------------------------------------------------------
------------------------------------------------------------
-------S----------------------------------------------------
------------------------------------------------------------
------------------------------------------------------------
------------------------KAKELKDRHRDFPDVIS--GAYIIEVIPDTPAEAGG
LKENDVIISINGQSVVSANDVSDVIKRE-STLNMVVRRGNEDIMITVIPE----EI----
--DP
>Latimeria_chalumnae-ENSLACP00000002935.1-HTRA4
M---------------------------------------------------M-------
-----------------------------------------------W------------
------A----------------V--------------------------C---------
FVL-P--L----VLVE----GR-VL-KR-QAPCPQLCDESRCP-PPAAQPCWAG------
-E--VRD-QCGCCLVCASREGELCGD----R---G----Y-GVCGEGLNCVYTGG-----
------------R--R--RSRG-TCVCSS-A---EPVCGSDGHT-YSNVC---------R
LKE-------------------------------------QTRRAQLS-HSPPVILIQRG
PCES---G--S-Q-HPDSLRYKFNFIADVVERIAPAVVHLELF-RR--LPF----TNREV
SISSGSGFVVSEDGMIVTNAHVLSN-------KQ---KIKVELKNGAQYDAKVLDADQKL
DIALIKI-E---------------------PE-----------------------SPLPV
LLLGRSSDLRPGEFVVAVGSPFSLQNTVTTGIVSTAQRGGKELGLKD---SDME-YIQTD
AIIN--YGNSGGPLLNLDGE--VIGINTLKVTAGISFAIPSDKIRQFLAESHD---RQL-
K--G-----------K--------------------TLPKKKYMGVRMLQLSS-------
------------------------------------------------------------
------------------------------------------------------------
------------------------------------------------------------
-------S----------------------------------------------------
------------------------------------------------------------
------------------------------------------------------------
```

```
------------------------LIHDLKSHDQDFPDVNS--GVYIFEVIPGTAAEGAG
LKDRDVIISINGKTISSTEDVSEAVKNS-DTLTVVVRRGKEDVVLTVVPE----E-----
--ID
>Latimeria_chalumnae-ENSLACP00000011379.1-HTRA1
M---------------------------------------------------M-------
-----------------------------------------------W------------
------P----------------V--------------------------L--------L
C-V-CWVLAS--FSAE----AR-ISKRY-VVGCPERCDPALCP-PLP-TDCPSG------
-Q--TLD-QCGCCSVCAAGEGEPCGG----SGRLG----E-PLCGDGLECSVSAG---VA
------ASLTVRR--R--GSPG-LCVCKS-A---EPVCGSDGKT-YRTVC---------E
LKS-------------------------------------ASSRAEKL-QQPPVILIQRG
ACGQ---G--Q-E-DPNSLRYKYNFIADVVEKIAPAVVHIELF-RK--LPF----SKREI
AVASGSGFIVSEDGLIVTNAHVVTN-------KH---RVKVEVKNGATYDAKIKDVDEKA
DIALIKI-D---------------------PK-----------------------GKLPV
LLLGHSADLRPGEFVVAIGSPFSLQNTVTTGIVSTTQRGGKELGLRN---SDMD-YIQTD
AIIN--YGNSGGPLVNLDGE--VIGINTLKVTAGISFAIPSDKIKEFLTESHD---RQS-
K--G-----------K--------------------TVNKKKYIGVRMMSLTP-------
------------------------------------------------------------
------------------------------------------------------------
------------------------------------------------------------
-------S----------------------------------------------------
------------------------------------------------------------
------------------------------------------------------------
------------------------LAKELKERQKDFPDVTS--GAYIVEVISKTPAAFAG
LKENDVIISINSQSIRSASDVSDVIKRE-NTLNVVVRRGNEDIILTVIPE----DI----
--EP
>Latimeria_chalumnae-ENSLACP00000012082.2-HTRA3
M---------------------------------------------------Q-------
-----------------------------------------------P------------
------L----------------A--------------------------L--VLL----
LL------------YA----LGLCG-AQ-PAKCPARCDVSTCA-S-P--SCPSG------
-Y--VPD-RCNCCLICAAGEGDACGR----K---D----D-PPCGDGLQCKHPAG-----
------------K--R--FGKG-VCQCKV-T---YKVCGSDGKT-YDNVC---------Q
MKA-------------------------------------VSRKALQQ-GLPAVIQLQKG
ACEL---G--H-Q-HSNSPRYKFNFIADVVEKIAPAVVHIELF-LR--HPL----FGRNV
PLSSGSGFVIADTGLIVTNAHVVSSSN-AVSGRQ---QLKVQLHNGDTYEAAIKDIDKKS
DIATIKV-N---------------------PK-----------------------KKLPV
LLLGHSTDLRPGEFVVAIGSPFALQNTVTTGIVSTAQRDGKELGLRD---SDMD-YIQTD
AIIN--YGNSGGPLVNLDGE--VIGINTLKVTAGISFAIPSDRITQFLAESHD---KES-
K------------------------------------EVKKRFIGIRMLTITP-------
------------------------------------------------------------
------------------------------------------------------------
------------------------------------------------------------
```

```
-------A----------------------------------------------------
------------------------------------------------------------
------------------------------------------------------------
------------------------LVEELKLSNPDFPDVSS--GIYVHEVVPNSPAQRGG
IKDGDIIVKVNGRPLLTTSDLQEAVMKE-TPLLLEVRRGNDDLLFNIEPE-----I----
--LM
>Latimeria_chalumnae-ENSLACP00000013119.1-HTRA2
L-----------------------------------------------PAVQA-------
------------------------------------------------------------
------------------------------------------------------------
------------------------------------------------------------
------------------------------------------------------------
------------------------------------------------------------
------------------------------------------------------------
---A---S--P-F-RPDSPRYKYNFIADVVDKAAPGVVYIEIL-GR--HPF----SGREV
PISNGSGFVVSEDGLIVTNAHVVAN-------KR---RVRVKLANGEVYNAVVDDVDQVA
DLAILKI-E---------------------AK-----------------------HPLPA
LLLGHSSDVRQGEFVVAMGSPFALQNTITSGIVSSAQRGSRELGLSH---SNMD-YIQTD
AAID--FGNSGGPLLNLDGE--VIGINTMKVTAGISFAIPSDRLKEFLNRAKT---KQR-
SWFS-----------S--------------------SEMKRRYIGVMMLTLTPKRCIPHC
STLPLGSSVLVSKFKKIYIFLYIYIIICSDNKWAGLNNRNSFTVEGIEPRESQIAHTGIQ
VISAFPILRLKSLQLISFQFKLLVSPFYLLNSLTCKGGGGSGKKQYRNNSSVQSFKTTNR
QEVVGEENQEMGKIQGRLWDSKHKSVRSIFCFLFVFLKRKKKKKKRYLFNLDKKRDTLFL
LFIFLYPS----------------------------------------------------
------------------------------------------------------------
------------------------------------------------------------
------------------------ILAELKLRDPGFPDVSH--GVLIHKVIIGSPAYHAG
LKPGDIILKINEKTVQTAEHIYDAVRSE-QNLTMVVQRGHEVLMMSVTPE-----V----
--TE
>Lepisosteus_oculatus-ENSLOCP00000002449.1-si
I---------------------------------------------------A-------
-----------------------------------------------N------------
------M----------------A------------------------------------
--------------------ASSRLLRL-FRNCLQ---DSRFPLRAL-GHTPGD------
-Q--VRE-KTNN------------------------------------------------
---------------------------------------------WSNGC---------H
RTGRYRTAWVIGLGIGGAALLYSRADSEDQSSPGTSPLGIRFTGASLH-N-----LLPVA
RCAT------P-V-KPDSPRYTYNFIADVVEKSAPAVVYIEIV-GR--HPY----TRREI
PISNGSGFIISKDGLIVTNAHVVAN-------KR---GVRVKLANGETYNAVVQDVDQVA
DVATIKI-N---------------------AK-----------------------HPLPT
LPLGVSAEVRQGEFVVAMGSPFALRNTITSGIVSSVQRGSRELGLSN---TNMD-YIQTD
AAID--FGNSGGPLINLDGE--VIGINTMKVTAGISFAIPSDHVKHFLNSTEE---RKK-
SWFG-----------E--------------------SEAKRRYVGVMMLTLTP-------
```


-------SYKVINTMLIPLIDLSLFFHIEILLYILLNQIGIFHFCTYDAKLFLKCKQNIA
IACIANNISSITVFISVSVKYLFLALCCEIMALYFLLYLFPPFFPITSILTGHSFSFLLG
CHECPAAADSATVMGGDSTEGFANSAGSLLMQSVLVVGQMLVSPTGNASNRNRPVLPGSQ
AAWCGVSFADRKEVRTAFCCLFPSIIAELKLRDPTFPDVTH--GVLIHRVIMGSPAYRAG
VKPGDVVLEINGKQVKTAEEIYNAVRTS-DSINMVVKRQDDLLILHMTPE-----V----
--TE
>Lepisosteus_oculatus-ENSLOCP00000010074.1-htra1b
I---A-------
---F----SFIYLLLL
AFTMLWP----------------L--------------------------F---------
C-A-GVLLVP--LIAE----AR-ISKRY-VIGCPDRCDKSQCP-QIP-ADCLAG------
-D--VLD-QCDCCPVCAAGEGEPCGG----SGRLG----D-PVCAEGLECSVSDG---VA
------YSATVRR--R--GKTG-TCVCKS-S---EPVCGSDGVS-YRNIC---------E
LKR-------------------------------------VSSGAQKL-QQPPVIFIQRG
ACGQ---GTAQ-E-NPDSLRHRYNFIADVVEKIAPAVVHIELY-RK--MVF----SKREV
AVASGSGFVVSEDGLIVTNAHVVAN-------KH---RVKVELKSGATYDAKIKDVDEKA
DIALIEI-D---------------------VP-----------------------MKLPV
LLLGRSADLRPGEFVVAIGSPFSLQNTVTTGIVSTTQRGGKELGLRN---SDMD-YIQTD
AIIN--YGNSGGPLVNLDGE--VIGINTLKVTAGISFAIPSDKIRQFLAESHD---RQA-
K--G-----------K--------------------TAPKKKYIGVRMMSLTP-------

-------S--

------------------------LAKDLKERQKDFPDVTS--GAYVIEVIAKTPAAVGG
LKEHDVIISINGQRISSASDVSDAIKRD-STLRLVVRRGNEDTILTIIPE----EI----
--DP
>Lepisosteus_oculatus-ENSLOCP00000012604.1-htra3a
M---K-------
---L------------
------F----------------L--------------------------F--GGV----
LLV------------I----QEFIDAEP-RPKCPSRCDVSRCP-S-P--SCPSG------
-Y--VPD-RCNCCLVCAHGEGDPCGR----K---D----D-LPCGDGLECKHPAG-----
------------K--R--LAKG-VCQCKL-A---YKVCGNDGKT-YGNVC---------Q
LKA-------------------------------------MSRKALQQ-GLPAIIQVQKG
PCES---G--P-Q-HPNSPRYKFNFIADVVEKIAPAVVHIELF-IR--HPL----FGRNV
PLSSGSGFIMTETGLIVTNAHVVTSTT-AVSGRQ---QLKVQMHNGDTYEATIKDIDKKS
DIATIKV-N---------------------PQ-----------------------KKLPV

```
LLLGQSADLRPGEFVVAIGSPFALQNTVTTGIVSTAQRDGKELGLRD---SDMD-YIQTD
AIIN--YGNSGGPLVNLDGE--VIGINTLKVTAGISFAIPSDRITRFLNESHD---KHS-
K--E-----------L--------------------KAVKKRFIGIRMLTITP-------
------------------------------------------------------------
------------------------------------------------------------
------------------------------------------------------------
-------G----------------------------------------------------
------------------------------------------------------------
------------------------------------------------------------
------------------------LVEELKQQDSDFPDVSS--GIYVHEVVPNSPAQKGG
IKDGDIIVKLNGRPLLSTGDLQEALMNE-SPLLLEA-------------------S----
--LC
>Lepisosteus_oculatus-ENSLOCP00000018588.1-htra4
I---------------------------------------------------S-------
-----------------------------------------------QKLRTLILMRSVI
NDIMTIK----------------L--------------------------L--------A
LIL-L--T----SVVH----AR-LLKKR-QTLCPEICDISRCP-PVP-DSCFYG------
-E--VKD-DCGCCDICAAGEGDLCGG----R---G----L-GTCGEGMVCVYPPG-----
------------K--R--RHRG-TCVCAA-T---EPVCGSDGRT-YPSIC---------R
LRA-------------------------------------ENRRAELN-QTPPVILIQKS
PCDS---G--P-Q-NPDSLRYKFNFIADVVDKIAPAVVHLELF-RR--LPF----TNQEI
PVSSGSGFIVSEDGWIVTNAHVLTN-------KQ---RIKVELKSGAQYDAKVKDVDQKL
DIALIKI-E---------------------PD-----------------------SHLPV
LLLGRSADLRPGEFVVAVGSPFSLQNTVTTGIVSTTQRGGRELGLKD---SDME-YIQTD
AIIN--YGNSGGPLVNLDGE--VIGINTLKVTAGISFAIPSDRIRQFLAESYD---RQI-
K--G-----------K--------------------TLPKKKYMGVRMLQLSP-------
------------------------------------------------------------
------------------------------------------------------------
------------------------------------------------------------
-------N----------------------------------------------------
------------------------------------------------------------
------------------------------------------------------------
------------------------LIRELKDRDKDFPDVSM--GVYVFEVIPGTAAASSG
MKDHDVIISINNQSVKTTEDVSEAVKSS-STLSVVVRRGNEDIMLTVVPE----E-----
--ID
>Monodelphis_domestica-ENSMODP00000005932.3-HTRA2
R---------------------------------------------------A-------
------------------------------------------------------------
------------------------------------------------------------
------------------------------------------------------------
------------------------------------------------------------
----------------------------------------RRWAAAAMGAGAAAALLLLW
GSG-------------------------------------GR--------GPPAVL----
```

-AAV---P--TPP-SSSSPRRLYNFIADVVEKTAPAVVYIEIL-GR--HPF----SGREV
PISNGSGFIVASDGLIVTNAHVVAD-------RR---RVRVRLPSGETYEATVTAVDPVA
DIATLRI-P---------------------TK-----------------------EPLPT
LPLGRSAEVRQGEFVVAMGSPFALQNTITSGIVSSAQRRARDLGLPQ---PNVE-YIQTD
AAID--FGNSGGPLVNLDGE--VIGVNTMKVTAGISFAIPSDRLREFLQRGGK---KSSW
F--G-----------T--------------------SESKRRYIGVMMLTLTP-------

-------S--

------------------------ILAELQLREPSFPDVQH--GVLIHKVILGSPAHRAG
LRPGDIILCIGDRLVKSAEDVYEAVRTQ-AKLAV--------------------------
---Q
>Monodelphis_domestica-ENSMODP00000010233.3-HTRA1
M---P-------
---V------------
------R----------------G--------------------------LGAA-LLPFL
LLL-LLSPVPGASQLS----RS-RYEAS-PVGCPERCDRARCA-PPP-DNCEGG------
-N--VPD-ACGCCQVCGAKEGEACGG----G---AG--GE-SPCGEGLQCVVPFG---VA
------ASATVRR--R--AQAG-LCVCAS-S---EPVCGSDAKT-YGNLC---------Q
LKA-------------------------------------ASRRSEKL-GQPPVIVIQRG
ACGQ---G--Q-E-DPNSLRHKYNFIADVVEKIAPAVVHIELF-RK--LPF----SKREI
PVASGSGFIVSEDGLIVTNAHVVTN-------KH---RVKVELKNGATYEAKIKDVDEKA
DIALIKI-D---------------------YQ-----------------------GKLPV
LLLGRSSELRPGEFVVAIGSPFSLQNTVTTGIVSTTQRGGKELGLRN---SDMD-YIQTD
AIIN--YGNSGGPLVNLDGE--VIGINTLKVTAGISFAIPSDKIKKFLTESHD---RQA-
K--G-----------K--------------------AVTKKKYIGIRMMSLTS-------

-------S--

------------------------KAKELKDRHRDFPDVLS--GAYIIEVIPDTPAEAGG
LKENDVIISINGKSVVSANDVSDVIKKE-NTLNMVVRRGNEDIMITVIPE----EI----
--DP
>Monodelphis_domestica-ENSMODP00000010236.3-HTRA3
M---L-------
---P------------
------S----------------T--------------------------F--L-LCALG
LL------------AL----LNPCQ-AE-PLACPPRCDVSKCP-S-P--SCPGG------

```
-Y--VPD-RCNCCLVCAAAEGDACGR----K---D----D-PPCGDSLECGHPAG-----
------------K--R--FAKG-VCQCKL-T---YQVCGSDGRT-YDNVC---------R
LKA-------------------------------------ASRKALQQ-GLPAVIQIQKG
ACES---G--H-Q-QFTSPRYKFNFIADVVEKIAPAVVHIELF-LR--HPL----FGRNV
PLSSGSGFIISESGLIVTNAHVVSSTN-AISGRQ---QLKVQLQSGDTYEAMIKDIDKKS
DIATIKI-N---------------------PK-----------------------KKLPV
LLLGHSTDLRPGEFVVAIGSPFALQNTVTTGIVSTAQRDGKELGLKD---SDMD-YIQTD
AIIN--YGNSGGPLVNLDGE--VIGINTLKVAAGISFAIPSDRITRFLTESYD---KQN-
K------------------------------------DVKKRFIGIRMRTITP-------
------------------------------------------------------------
------------------------------------------------------------
------------------------------------------------------------
-------V----------------------------------------------------
------------------------------------------------------------
------------------------------------------------------------
------------------------LVEELKDNNPDFPDVSS--GIYVHEVVPNSPSQRGG
IKDGDIIVKVNGRPLKNSSELQEAVMKE-SPLLLEVRRGNDDLLFNIEPE-----V----
--VM
>Monodelphis_domestica-ENSMODP00000013256.2-HTRA4
E---------------------------------------------------V-------
----------------------------------------------EM------------
------A---------------VW----------------------ALGAGKSR-PLPFL
LLL-L--STVAMPWIQ----AR-P-PWT-PTSCPPTCELTRCP-PLP--DCPMG------
-APPQPD-RCNCCLVCAAGEGEMCGV----P---W----S-RPCAPGLRCRVTRT-----
----------LKN--R--GQLG-ICVCGA-S--EVPVCGNDGRS-YQSLC---------A
LRA-------------------------------------ENRVARLR-GGLQAVPVEKG
NCG-----------NPGELDYGVSLLSGPLLSPVPAPVGLSIG-PH--SPFWVSLSSEYT
HASSGSGFIVSEDGLIVTNAHVLTN-------RQ---RINVELQNGAKYEATVKDVDQKT
DLALIKI-E---------------------PE-----------------------TDLPV
LFLGRSSNLQAGEFVVALGSPFSLQNTVTAGIVSTTQRGGKELGLKD---SDMD-YIQTD
AIIN--HGNSGGPLLNLDGE--VIGINTLKVTAGISFAIPSDRIRQFLAEFYQ---RQL-
K--G-----------K--------------------VLSQKKYLGLRMLPLSM-------
------------------------------------------------------------
------------------------------------------------------------
------------------------------------------------------------
-------S----------------------------------------------------
------------------------------------------------------------
------------------------------------------------------------
------------------------LLQEMKNQDPDFPEVSS--GVFVYEVIQGTPAASSG
MRNHDVITSINGQPVTSITDVIEAVKES-DSISLVVRRRNEDVVLTIIPE----I-----
--IN
>Mus_musculus-ENSMUSP00000006367.7-Htra1
M---------------------------------------------------Q-------
```

---S------------
------L----------------R----------------------------TT-LLSLL
LLL-L--AAP--SLALPSGTGR--SAPA-ATVCPEHCDPTRCA-PPP-TDCEGG------
-R--VRD-ACGCCEVCGALEGAACGL----Q--------E-GPCGEGLQCVVPFG---VP
------ASATVRR--R--AQAG-LCVCAS-S---EPVCGSDAKT-YTNLC---------Q
LRA-------------------------------------ASRRSEKL-RQPPVIVLQRG
ACGQ---G--Q-E-DPNSLRHKYNFIADVVEKIAPAVVHIELY-RK--LPF----SKREV
PVASGSGFIVSEDGLIVTNAHVVTN-------KN---RVKVELKNGATYEAKIKDVDEKA
DIALIKI-D----------------------HQ----------------------GKLPV
LLLGRSSELRPGEFVVAIGSPFSLQNTVTTGIVSTTQRGGKELGLRN---SDMD-YIQTD
AIIN--YGNSGGPLVNLDGE--VIGINTLKVTAGISFAIPSDKIKKFLTESHD---RQA-
K--G-----------K--------------------AVTKKKYIGIRMMSLTS-------

-------S--

------------------------KAKELKDRHRDFPDVLS--GAYIIEVIPDTPAEAGG
LKENDVIISINGQSVVTANDVSDVIKKE-NTLNMVVRRGNEDIVITVIPE----EI----
--DP
>Mus_musculus-ENSMUSP00000081044.4-Htra4
M---S-------
---F------------
------Q----------------R----------------------LWAVRTQF-LLLWL
LLP-A--V--PVPWAE----AR-R-SRV-SLPCPDACDPTRCP-TLP--TCSAG------
-LAPVPD-RCGCCRVCAAAEGQECGG----A---R----G-RPCAPRLRCGAPFS---R-
---------DPSG--G--AWLG-TCGCAE-GAEDAVVCGSDGRT-YPSLC---------A
LRK-------------------------------------ENRAARQR-GALPAVPVQKG
ACEE--AG--T-T-RAGRLRRKYNFIAAVVEKVAPSVVHLQLF-RR--SPL----TNQEI
PSSSGSGFIVSEDGLIVTNAHVLTN-------QQ---KIQVELQSGARYEATVKDIDHKL
DLALIKI-E----------------------PD----------------------TELPV
LLLGRSSDLRAGEFVVALGSPFSLQNTVTAGIVSTTQRGGRELGLKN---SDID-YIQTD
AIIN--HGNSGGPLVNLDGD--VIGINTLKVTAGISFAIPSDRIRQFLEDYHE---RQL-
K--G-----------K--------------------APLQKKYLGLRMLPLTL-------

-------N--

------------------------LLQEMKRQDPEFPDVSS--GVFVYEVIQGSAAASSG
LRDHDVIVSINGQPVTTTTDVIEAVKDN-DFLSIIVLRGSQTLFLTVTPE----I-----

```
--IN
>Mus_musculus-ENSMUSP00000084910.3-Htra3
M---------------------------------------------------Q-------
-----------------------------------------------A------------
------R----------------A--------------------------L--L---PAT
LAI-L--ATL----AV----LALAREPP-AAPCPARCDVSRCP-S-P--RCPGG------
-Y--VPD-LCNCCLVCAASEGEPCGR----P---L----D-SPCGDSLECV---------
--------------------RG-VCRCRW-T---HTVCGTDGHT-YADVC---------A
LQA-------------------------------------ASRRALQV-SGTPVRQLQKG
ACPS---G--L-H-QLTSPRYKFNFIADVVEKIAPAVVHIELF-LR--HPL----FGRNV
PLSSGSGFIMSEAGLIVTNAHVVSSSS-TASGRQ---QLKVQLQNGDAYEATIQDIDKKS
DIATIVI-H---------------------PK-----------------------KKLPV
LLLGHSADLRPGEFVVAIGSPFALQNTVTTGIVSTAQRDGKELGLRD---SDMD-YIQTD
AIIN--YGNSGGPLVNLDGE--VIGINTLKVAAGISFAIPSDRITRFLSEFQN---KHV-
K------------------------------------DWKKRFIGIRMRTITP-------
------------------------------------------------------------
------------------------------------------------------------
------------------------------------------------------------
-------S----------------------------------------------------
------------------------------------------------------------
------------------------------------------------------------
------------------------LVEELKAANPDFPAVSS--GIYVQEVVPNSPSQRGG
IQDGDIIVKVNGRPLADSSELQEAVLNE-SSLLLEVRRGNDDLLFSIIPE-----V----
--VM
>Mus_musculus-ENSMUSP00000087073.6-Htra2
M---------------------------------------------------A-------
-----------------------------------------------A------------
------L----------------KAGRGANWSLRAWRALGGIFWRKPPLLA--------P
D-L-RALLTS--GTPD----SQ-IWMTYGTPSLPAQVPEGFL---AS-RADLTS------
-R--TPDLWARLNVG---------------------------------------------
--------------------TS-GSSDQE-A---RRSPGSRRREWLAVAVGAGGAVVLLL
WGW-------------------------------------GR--------GLSTVL----
-AAV---P--A-P-PPTSPRSQYNFIADVVEKTAPAVVYIEIL-DR--HPF----SGREV
PISNGSGFVVASDGLIVTNAHVVAD-------RR---RVRVRLPSGDTYEAMVTAVDPVA
DIATLRI-Q---------------------TK-----------------------EPLPT
LPLGRSADVRQGEFVVAMGSPFALQNTITSGIVSSAQRPARDLGLPQ---NNVE-YIQTD
AAID--FGNSGGPLVNLDGE--VIGVNTMKVTAGISFAIPSDRLREFLHRGEK---KNSW
F--G-----------T--------------------SGSQRRYIGVMMLTLTP-------
------------------------------------------------------------
------------------------------------------------------------
------------------------------------------------------------
-------S----------------------------------------------------
------------------------------------------------------------
```

```
------------------------------------------------------------
------------------------ILIELQLREPSFPDVQH--GVLIHKVILGSPAHRAG
LRPGDVILAIGEKLAQNAEDVYEAVRTQ-SQLAVRIRRGSETLTLYVTPE-----V----
--TE
>Petromyzon_marinus-ENSPMAP00000002921.1-si
M---------------------------------------------------P-------
------------------------------------------------------------
------------------------------------------------------------
------------------------------------------------------------
------------------------------------------------------------
------------------------------------------------------------
------------------------------------------------------------
---E---P--N-H-SVESRRARYNFIADVVQRAAPAVVHIEIF-KS--PPS----YARAA
PVSSGSGFLVSDEGLIVTNAHVVGGGG----ATR---VVMVTLRGGRSYEAEVTDVDTKV
DIATIRI-H---------------------PQ-----------------------HPLAV
LQLGRSSELRPGEFVVAIGSPFALQNTVTTGM-STAQRGGRELGLHN---SKRDTYWIAK
HTKH--YGNSGGPLVNLVSD--V-------------------------------------
------------------------------------------------------------
------------------------------------------------------------
------------------------------------------------------------
------------------------------------------------------------
------------------------------------------------------------
------------------------------------------------------------
------------------------------------------------------------
------------------------------------------------------------
------------------------------------------------------------
----
>Tetraodon_nigroviridis-ENSTNIP00000006685.1-HTRA3
K---------------------------------------------------E-------
-----------------------------------------------R--------LRVT
ERSSMNL----------------L--------------------------L--RAP---L
LLY------------L----LDVLGAQP-EAKCPTRCDVSACP-S-P--GCPGG------
-Y--VPD-RCNCCLVCAHGEGEACGR----K---D----D-LPCGDGLECQHPAG-----
------------R--R--LAPG-VCRCKL-S---SEVCGSDGKT-YGNAC---------Q
LQA-------------------------------------SSRKALQQ-GQPGLRQVRRG
PCESG-SG--S-S-RASSPRYKFNFIADVVEKIAPAVVHIELF-LR--HPL----FGRTV
PLSSGSGFLVSENGLIVTNAHVVSSTS-PGTAQQ---HLKVQMQNGDTYEASIKDIDKKS
DIATIKV-E---------------------PQ-----------------------VKLPV
LFLGQSADLRPGEFVVAIGSPFALQNTVTTGIVSTAQRDGRELGLRD---SDMD-YVQTD
AIIN--YGNSGGPLVNLDGE--VIGINTLKVAAGISFAIPSDRITQFLSHALQ---RHG-
K--D-----------A--------------------KSAKKRFIGIRMLTVTP-------
------------------------------------------------------------
------------------------------------------------------------
```

-------G--

------------------------LMEELKQQNPDFPDIGG--GIYVHGVVPLSPADKGG
IKEGDVLVKLNGRPLASTADLQGALQEE-AALLLEVRRGNDDLLFNIQPD----LI----
--LQ
>Tetraodon_nigroviridis-ENSTNIP00000014844.1-htra1b
R--LISE-------
---S------------
------F----------------H----------------------------SP-IMRLG
LLL-C-ALAM--LVAE----AR-IVKRL-AIGCPEKCDKSQCA-PIP-ADCLAG------
-D--ALD-RCDCCPVCASGEGEQCGG----S---G----D-RDCAEGMECLVADG---VE
------VSATVRK--R--GAAG-LCVCTS-S---EPVCGSDGVS-YRNIC---------E
LKR-------------------------------------VSNRAVKL-QRPPVILIQRG
ACGE---G--Q-Q-NPDSLRYKYNFIADVVEKIAPAVVHIELY-RK--TIF----TKREV
AVASGSGFVVSEDGLIVTNAHVVAN-------KH---RVKVELKSGATFDAKIRDVDEKA
DIALIKI-D---------------------AP-----------------------MKLPV
LLLGRSADLRPGEFVVAIGSPFSLQNTVTTGIVSTTQRGGKELGLRN---SDMD-YIQTD
AIIN--YGNSGGPLVNLDGE--VIGINTLKVTAGISFAIPSDKIRQFLAESHD---RQA-
R--G-----------Q--------------------TVQKKKYIGIRMMSLTP-------

-------T--

------------------------LAKDLKERQSDFPDVTS--GVYVIEVISRTPAERAG
LHEKDVIISINGEPISLASDVSDAIKGG-DALRMVVRRGNEDVILTVVPE----EI----
--DP
>Tetraodon_nigroviridis-ENSTNIP00000020138.1-htra1a
V---A-------
---Q------------
------L----------------P----------------------------SA-NMFWW
LSL-C-AVAA---LAE----AQ-SSGRF-VVGCPARCDKSTCP-RPP-ADCAAG------
-Q--TLD-ACRCCPVCASGEGEACGG----TGKLG----D-PVCGDGLECSVPGG---VA
------HSGTVRR--R--SRSG-TCTCKA-S---EPVCGSDGVS-YRNTC---------E
LRR-------------------------------------ASRRAQRL-LQPPVLFVQRG
ACGK---A--Q-D-NPDSPRHKYNFIADVVEKIAPSVVHIELF-RK--MTY----SKREV
PVASGSGFVVSEDGQIVTNAHVVAN-------KH---RVKVELQSGGSYDAKIQDVDERS
DIALIKI-A---------------------AP-----------------------TKLPV
LLLGRSSDLRPGEFVVAIGSPFSLQNTVTTGIVSTTQRGGRELGLQN---SDMD-YIQTD
AIIN--YGNSGGPLINLDGE--VVGINTLKVTAGISFAIPSDKIREFLAESYD---RQS-

R--G-----------R--------------------TAAKKKYIGVRMMTLTP-------

-------A--

------------------------LAKELKTQHRDFPDITS--GAYVMEVIAKTPAAVGG
LKEHDVIISINGQRISSASDVSAAIKKD-NKLSVVVRRGNEDLILTVVPV----EI----
--DP
>Tetraodon_nigroviridis-ENSTNIP00000020475.1-si

---------------PDSPRYKYNFIADVVEKSTPAVVYIEILGSR--HPF----SGREI
TVSNGSGFIISNDGLIVTNAHVVAN-------KR---GVRVKLNNGDVYDAAVQEVDQVA
DIATIKI-SVKVTKDRRSSPACVMELLFLHPQ-----------------------KPLPT
LPLGRSAEVRQGEFVVAMGSPFALRNTITSGIVSSAQRGSRELGLSN---SNMD-YIQTD
AAID--FGNSGGPLINLDGE--VIGINTMKVTAGISFAIPSDRLRTFLDQAEK---KKS-
SWFR-----------D--------------------SDPRRRYIGVMMLTLTP-------

-------S--

------------------------IIAELKLRDGSFPEVTH--GVLIHRVIMGSPADRAG
LTAGDIVVEINGAKVNTSEEIYQAVRSG-DQITMLVQRGDGLLRLRVTPE-----Y----
--TE
>Xenopus_tropicalis-ENSXETP00000033189.3-htra4
M---S-------
---P------------
------P----------------W--------------------------S--L--LPIF
FLF-L--SAS----AA----PGI-SRRQ-TPNCPRVCELARCP-RIQ-MPCQAG------
-E--VRD-SCGCCPVCGASEGEACGH----R---G----G-APCGEGMECVLPAV-----
---------GGSA--R--RRIG-VCVCST-S---EPVCGSDGRT-YRNLC---------H
LKA-------------------------------------ENRRARLL-NSPPAIHIQKG
PCDT---G--S-H-YPDSLRYKFNFIADVVQKIAPAVVHLELF-RR--SPF----TGQEM
AVSSGSGFIVSDDGLIVTNAHVLTN-------KQ---RIKVEVKDGAHYDAKIKDIDQKL

```
DIALIKI-D---------------------PD-----------------------APLPV
LMLGRSADLRPGEFVVALGSPFSLQNTVTTGIISTTQRGGKELGLKD---SDME-YIQTD
AIIN--YGNSGGPLVNLDGE--VIGINTLKVTAGISFAIPSDRIRQFLAESHD---RQT-
K--G-----------K--------------------MLPKKKYMGVRMLQLSS-------
------------------------------------------------------------
------------------------------------------------------------
------------------------------------------------------------
-------N----------------------------------------------------
------------------------------------------------------------
------------------------------------------------------------
------------------------LIRELKTRDKDFPDVNA--GVYVFEVIPGTAAASAG
MKDHDVIISLNGKMVSSTEEVSEAVRNN-DTLSIVVRRGNEDIILNVVPE-----E----
--ME
>Xenopus_tropicalis-ENSXETP00000033992.3-htra1
L---------------------------------------------------S-------
-----------------------------------------------A------------
------F----------------G--------------------------M--A-M-LWL
AVL-L--TCG----AP----AAL-LPTS-GVGCPSRCDPASCA-PAP-TNCPAG------
-E--TAL-RCGCCPVCAAAEWERCGE---GP---E----D-PLCASGLRCVKNG------
-------------------GVA-RCQCPS-N---LPVCGSDGKT-YPSLC---------R
LQA-------------------------------------ESKAAQGK-GSAAIIPIQRG
DCQQ---G--Q-R-DPDSPRYKYNFIADVVEKIAPAVVHIELF-RM--LPF----FKREV
PAASGSGFIVSEDGLILTNAHVVTN-------KH---RLKVERSDGSTYDAQIIDVDEKA
DIALIKI-K---------------------AK-----------------------GKLPV
LLLGRSEDLRPGEFVVAIGSPFSLQNTVTTGIVSTAQRGGKELGLRN---SDMD-YIQTD
AIIN--YGNSGGPLVNLDGE--VIGINTLKVTAGISFAIPSDKIRKFLAESHN---RQS-
T--G-----------Q--------------------GTKKKKYLGIRMMSLSQ-------
------------------------------------------------------------
------------------------------------------------------------
------------------------------------------------------------
-------G----------------------------------------------------
------------------------------------------------------------
------------------------------------------------------------
------------------------KLKELKEQVKDFPENTS--GAYIVEVIPDTPAEEAG
LKEGDIIISIGGKSVTSSSDVSDAIKKEGTTLHLVIRRGNEDIPISVTPK-----E----
-IEF
>Xenopus_tropicalis-ENSXETP00000062635.1-HTRA3
E------------------------------------TPQLSELPISLLLRMK-------
-----------------------------------------------L------------
------C----------------A--------------------------W--LPL----
CAL------------L----TLLSGATR-TGACPARCDVSRCP-S-P--ICPSG------
-Y--VPD-RCNCCLICAAGEGEPCGR----E---G----D-PPCGDSLQCKPPPG-----
------------M--R--GLKG-SCQCKH-T---NPVCANDGQT-YDNLC---------Q
```

LKA-------------------------------------ASRRALQK-GHSPVVLVQKG
ACQSASIG--H-Q-HPNSPRYKFNFIADVVEKIAPAVVHIELF-LR--HPL----FGRNV
PLSSGSGFIISDLGLIVTNAHVVSSSN-TVSGRQ---YLKVQLHNGDSYEATIKDIDKKS
DIATIKI-Y---------------------PR-----------------------KKLPV
LLLGHSADLRPGEFVVAIGSPFALQNTVTTGIVSTAQRDGKELGLRD---SDMD-YVQTD
AIIN--YGNSGGPLVNLDGE--VIGINTLKVAAGISFAIPSDRITKFMTESQD---KKY-
K--A-----------N--------------------NDGKKRFIGIKMLTITP-------

-------S--

------------------------FAEEKKLHNPDFPDVTS--GIYVHEVVPNSPAQRGG
IQDGDIIVKVNGRPLVTSGDLHEAVMNE-SPLLLEVRRGNDDMLFNLEPE-----V----
--SM
>Xenopus_tropicalis-ENSXETP00000063073.1-HTRA2

--PLPT
LRLGRSAEIRQGEFVVAMGSPFSLQNTITSGIVSSVQRGGHELGLAN---RDME-YIQTD
AAID--FGNSGGPLVNLGGQ--MIGSGAQTGSVEWSLGLGC--VLVLIGVLGH---RLG-
SLLG-----------S--------------------SEVKRRYIGVMMLTLTP-------

-------K--

------------------------ILAELKFRDPSFPDVSH--GVLIHKVIVGSPAHESG
LRAGDVVLEINAKRAMTSEDIYDAVHTH-PKLTMIVRRGSESLMVTVIPE-----C----
--TE
>Callorhinchus_milii-SINCAMP00000016221
M---G-------
---G------------
------S----------------A--------------------------V--S-LVTCC

```
WLLCA--GWG----WG----VAAQT-LD-PPGCPGRCDVSKCP-S-P--SCLHG------
-Y--VHD-SCHCCLVCAAGQGQGCGR----P---G----D-ATCGEGMKCVRAHR-----
------------K--RLPRAQG-VCRCKL-N---YRVCGSDGKT-YHNLC---------Y
LKL-------------------------------------VSRRALQL-GMPSVSQLHKG
SCET---G--E-S-HPNSPRYKFNFIADVVEKIAPAVVHIERF-LR--HPL----FQRSV
PLSSGSGFLISDSGLIVTNAHVVMSNNNLMSSRQ---QIKVQVHNGDNYEATITDIDKKS
DIATIKI-N---------------------SK-----------------------KKLPV
LLLGRSVDLRPGEFVVAIGSPFALQNTVTTGIVSSAQRDGKELGLRD---SDMD-YIQTD
AIIN--YGNSGGPLVNLDGE--VIGINTLKVTAGISFAIPSDRITQFLTDSHD---KQS-
K--V-----------K---------------------VTRKRYIGVKMLTITP-------
------------------------------------------------------------
------------------------------------------------------------
------------------------------------------------------------
-------D----------------------------------------------------
------------------------------------------------------------
------------------------------------------------------------
------------------------LMQELKQRHPDFPDIST--GIYIHEVIPDSPAQRRD
S------------------------------------------------------N----
--FH
>Callorhinchus_milii-SINCAMP00000005070
M---------------------------------------------------S-------
-----------------------------------------------Q------------
------I----------------G--------------------------L--------F
C---GLLCCW--LAAE----AR-VS----RQTCPEVCDTSTCP-PAP-RNCSFG------
-E--AKD-SCDCCSFCAAGEGDFCGG----WD-DH----H-GICGESFRCLYPNG-----
------------N--R---RKG-TCGCVH-G---DQVCGSDGQT-YKNIC---------T
LKA-------------------------------------MSKKQQEA-NIPPVILIQKG
PCDS---D--H-Q-HPDSMRFKYNFIADVVDKIAPAVVHLALV-RR--ASY----SNRDV
TVGSGSGFIVSEDGLIVTNAHVLNN-------KQ---RIKVELKNGEKYEAKVKDIDRKS
DIALIKI-K---------------------AN-----------------------SSLPV
LLLARSTDLRPGEFVVAIGSPFSLQNTVTTGIVSTTERGGKELGLKD---SDMD-YIQTD
AIIN--YGNSGGPLVNLDGE--VIGINTLKVTAGISFAIPAKRIQQFLAESYD---REL-
K--G-----------K--------------------SLPLKRYMGVRMLKLTP-------
------------------------------------------------------------
------------------------------------------------------------
------------------------------------------------------------
-------N----------------------------------------------------
------------------------------------------------------------
------------------------------------------------------------
------------------------LSRELKAHDKHFPDVSG--GVYIFEVMSGSPAQSAG
LKDRDVIISINGIPITSSEQVSEAVKNS-NSLSIGVRRSNGVLLITVVPE----NQVIKG
DKGK
>Callorhinchus_milii-SINCAMP00000002753
```

```
L---------------------------------------------------C-------
------------------------------------------------------------
------------------------------------------------------------
------------------------------------------------------------
------------------------------------------------------------
------------------------------------------------------------
----------------------------------------YY--------LQVTFD----
-FLA---G--Q-K-DPNSLRLKYNFIADVVDKIAPAVVHIELF-RR--SPF----SNREM
SVASGSGFIVSEDGLIVTNAHVVTN-------KH---RVKVELRNGATYNAKIKDIDEKA
DIALIKI-D---------------------TP-----------------------DKLPV
LLLGHSIDLRPGEFVVAIGSPFSLQNTVTTGIVSTTQRGGKELGLRN---SDMD-YIQTD
AIIN--YGNSGGPLVNLDGE--VVGINTLKVTAGISFAIPSDKIRQFLAESYD---RQS-
K--G-----------I--------------------TSVKKRYLGVRMITLSF-------
------------------------------------------------------------
------------------------------------------------------------
------------------------------------------------------------
-------S----------------------------------------------------
------------------------------------------------------------
------------------------------------------------------------
------------------------LAKELRDRHKDFPDINA--GAYIIEVLPRTPAAAGG
LRDNDVIISINNQLVTSASDVSEAIKKD-SSLSILVRRKNEDLRINVVTE----EI----
--EP
>Callorhinchus_milii-SINCAMP00000018559
------------------------------------------------------------
------------------------------------------------------------
------------------------------------------------------------
------------------------------------------------------------
------------------------------------------------------------
------------------------------------------------------------
------------------------------------------------------------
---------------------------------------CGLF-FR--HPF----SGREI
PVSNGSGFLVTQDGLIVTNAHVVAN-------KR---RVRVRLADGETYDATVQDVDQVS
DIATIKI-N---------------------PM-----------------------RPLPT
LGLGQSSDVRHGEFVVAMGSPFALQNTITSGIVSSVQRGSRELGMGH---SNME-YIQTD
ATID--FGNSGGPLVNLDGE--VIGVNTMKVTAGISFAIPSDRLREFLTLSQQ---RKS-
K--S--------------------------------------------------------
------------------------------------------------------------
------------------------------------------------------------
------------------------------------------------------------
------------------------------------------------------------
------------------------------------------------------------
------------------------------------------------------------
------------------------------------------------------------
```

```
------------------------------------------------------------
--EC
>Petromyzon_marinus-ENSPMAP00000002693x
M---------------------------------------------------I-------
-----------------------------------------------S------------
------WGAVAGWPLLVPPPLPPP--------------------------PLLLLL-VSL
VP-------------------CLLISAQ-EADPRP-------------------------
----------------GAQEGEPCLR----A---ASPRSP-RRCAAGLECEPLDA-----
--EEPRTGARLWG--S--RGKG-LCVCAA-LR-GVAVCASDGRT-YGSVC---------R
LRS-------------------------------------EQRRLGAG-AGGSLFPIFNG
SCET---G--A-P-NPDSVRERLNFIANVTERARRAVVHVELL-AR--HLL----FDRNV
SVSSGSGFLVSEDGLILTNAHVVTH-------KRDLVAVKVQLTNGDTYEAWPLHVDMHA
NIAVIKI-N---------------------AK-----------------------QKLPV
LRLARSAELRPGEFVVAIGSPFKLQNTVTTGIVSTAQRGGQELGLDD---SDHD-YIQTD
AMVN-------------DGE--VVGINTLKVTAGISFAIPSDRIRELLLQRPD---TAA-
S--A-----------G--------------------RESAFESWGARTPARTS-------
------------------------------------------------------------
------------------------------------------------------------
------------------------------------------------------------
-------Q----------------------------------------------------
------------------------------------------------------------
------------------------------------------------------------
----------------------------VHP-----------------------RADGGG
NRE---------------------------------AAAAAAAAVDFIPGR--IHS----
--AA
>Petromyzon_marinus-ENSPMAP00000002696x
M---------------------------------------------------G-------
-----------------------------------------------S------------
------P----------------G--------------------------LHKA-LLPVA
FL-------------------LLALAGE-RGGCAE-------------------------
----------------RAGEGEPCVSVMALG---GPGPAP-RVCRAGLVCRPVVG---VA
GAQQQHQHQHGGK--R--RAKG-VCECKH-P---GPVCSSEGRT-FSSVC---------A
LRA-------------------------------------HGAKA-----------LHAG
PCPN---G--S-Q-PHKSLRDRFNFIADVVEKIVPAVVHIELY-SR--HPF----FNRNI
SVASGSGFMVSEDGVIVTNAHVVVN-------KR---YVKVQLHDGESYEASVVDADEKA
DIATIRI-K---------------------AK-----------------------QKLPV
LRLARSAELRPGEFVVAIGSPFKLQNTVTTGIVSTAQRGGQELGLED---SDAD-YIQTD
AIIN--VSASGEPA-GPGGF--VVGDDGARLPPRAYVSVASWTKRREMHGG---------
------------------------------------------------------------
------------------------------------------------------------
------------------------------------------------------------
------------------------------------------------------------
------------------------------------------------------------
```

```
------------------------------------------------------------
------------------------------------------------------------
------------------------------------------------------------
PRD-NFTSRVHGSGV-------------------VVKQAWHSVVERVEPKRCTDIV----
--GR
>Branchiostoma_floridae-C_1530089
M---------------------------------------------------T-------
-----------------------------------------------R------------
------T-----------------------------------------------------
------------------------------------------------------------
---------GFYLRTCSTSVGN-----------------------------NTSK-----
---------GYKY--L--RDSGIVVGCFA-W---GYVIGKFGVP-YIWPS---------T
DSA-------------------------------------DKEKSSIVKKVDAAEP-FYG
-------G--E-GYGGIPRSKQFNFIADVVEIASPAVVYIEIQ-GK--NPF----TGGRA
PTSNGSGFIVREDGLVVTNAHVVAN-------KR---YVKVRLQDGRLLDGVVTLVDQAA
DIAAVKIIN---------------------CN-----------------------TPLKT
VKLGNSSTLRPGEWVVAMGSPLSLSNTITAGVISSVQRGSRELGLRH---NDMD-YIQTD
AAIN--FGNSGGPLVNLDGE--VIGVNTMKVTTGISFAIPIDKVKEFLKNVEE---KEK-
A--QKGWFGRGQVAPP--------------------SPPKRRYLGVTMVTLTP-------
------------------------------------------------------------
------------------------------------------------------------
------------------------------------------------------------
-------N----------------------------------------------------
------------------------------------------------------------
------------------------------------------------------------
------------------------IIMELQERRTDFPDVRT--GVLVHRIIVGSPAYSAG
IRPGDVITSINGRQVTSARDIYDAVNSG-QQLNITCHRGRTVHHLQVTPE-----E----
--AD
```
